# Human LINE-1 restriction by APOBEC3C is deaminase independent and mediated by an ORF1p interaction that affects LINE reverse transcriptase activity

**DOI:** 10.1093/nar/gkt898

**Published:** 2013-10-05

**Authors:** Axel V. Horn, Sabine Klawitter, Ulrike Held, André Berger, Ananda Ayyappan Jaguva Vasudevan, Anja Bock, Henning Hofmann, Kay-Martin O. Hanschmann, Jan-Hendrik Trösemeier, Egbert Flory, Robert A. Jabulowsky, Jeffrey S. Han, Johannes Löwer, Roswitha Löwer, Carsten Münk, Gerald G. Schumann

**Affiliations:** ^1^Section PR2/Retroelements, Paul-Ehrlich-Institut, Paul-Ehrlich-Strasse 51-59, 63225 Langen, Germany, ^2^Department of Embryology, Carnegie Institution of Washington, 3520 San Martin Drive, Baltimore, MD 21218, USA, ^3^Division of Medical Biotechnology, Paul-Ehrlich-Institut, Paul-Ehrlich-Strasse 51-59, 63225 Langen, Germany, ^4^Clinic for Gastroenterology, Hepatology and Infectiology, Medical Faculty, Heinrich-Heine-University, 40225 Düsseldorf, Germany and ^5^Biostatistics Section, Paul-Ehrlich-Institut, Paul-Ehrlich-Strasse 51-59, 63225 Langen, Germany

## Abstract

LINE-1 (L1) retrotransposons are mobile genetic elements whose extensive proliferation resulted in the generation of ∼34% of the human genome. They have been shown to be a cause of single-gene diseases. Moreover, L1-encoded endonuclease can elicit double-strand breaks that may lead to genomic instability. Mammalian cells adopted strategies restricting mobility and deleterious consequences of uncontrolled retrotransposition. The human APOBEC3 protein family of polynucleotide cytidine deaminases contributes to intracellular defense against retroelements. APOBEC3 members inhibit L1 retrotransposition by 35–99%. However, genomic L1 retrotransposition events that occurred in the presence of L1-restricting APOBEC3 proteins are devoid of detectable G-to-A hypermutations, suggesting one or multiple deaminase-independent L1 restricting mechanisms. We set out to uncover the mechanism of APOBEC3C (A3C)-mediated L1 inhibition and found that it is deaminase independent, requires an intact dimerization site and the RNA-binding pocket mutation R122A abolishes L1 restriction by A3C. Density gradient centrifugation of L1 ribonucleoprotein particles, subcellular co-localization of L1-ORF1p and A3C and co-immunoprecipitation experiments indicate that an RNA-dependent physical interaction between L1 ORF1p and A3C dimers is essential for L1 restriction. Furthermore, we demonstrate that the amount of L1 complementary DNA synthesized by L1 reverse transcriptase is reduced by ∼50% if overexpressed A3C is present.

## INTRODUCTION

LINE-1 (L1) retrotransposon activity can cause disease by insertional mutagenesis, recombination, providing enzymatic activities for other non-long terminal repeat (non-LTR) retrotransposons, and perhaps by transcriptional over-activation and epigenetic effects [reviewed in (1,2)]. Since L1 elements were discovered as mutagenic insertions in 1988 (3), 96 disease-causing mutations in humans have been attributed to L1-mediated retrotransposition events [reviewed in (4)]. Recent reports also suggest that L1 endonuclease may have a general function in facilitating chromosomal breaks and genome instability (5,6). To limit such deleterious effects of retrotransposition, host genomes have adopted several strategies to curb the proliferation of transposable elements. Mechanistic strategies used by the host to restrict the mobilization of transposable elements include DNA methylation (7–9), small-RNA–based mechanisms (10–12), DNA repair factors (13,14) and L1 restriction by TREX1 DNA exonuclease (15) and members of the human APOBEC3 (*Apolipoprotein B mRNA Editing Enzyme Catalytic Polypeptide 3*, A3) protein family of cytidine deaminases [reviewed in (16)].

The human A3 protein family comprises seven members that include either one (APOBEC3A [A3A], APOBEC3C [A3C], APOBEC3H [A3H]) or two (APOBEC3B [A3B], APOBEC3D [A3D], APOBEC3F [A3F], APOBEC3G [A3G]) cytidine deaminase (CDA) domains containing a conserved zinc-binding motif (C/H)-X-E-X_23-28_-P-C-X_2-4_-C (17–20). Multiple A3 genes were reported to be expressed constitutively in most types of cells and tissues (21). A3 proteins can restrict the replication of various retroviruses and LTR retrotransposons by deaminating cytidines during the first-strand complementary DNA (cDNA) synthesis, which leads to either cDNA degradation or the integration of a mutated provirus or LTR retrotransposon, respectively (22,23). All A3 enzymes exhibit specificity for single-stranded DNA. Since the reaction product is deoxyuridine, A3 activity results in DNA peppered with C→U substitutions, referred to as hypermutants. The degree of editing can range from a few cytidine targets per kilobase to ∼90% of all cytidine residues (24–31). All A3 enzymes preferentially edit single-stranded DNA when the edited base is 5′-flanked by thymidine or cytidine, i.e. TpC and CpC [summarized in (32)]. However, recent studies have provided evidence suggesting that A3 proteins can inhibit retroviruses by editing-independent processes (33). For instance, antiviral activities of wild-type (WT) and catalytically inactive A3F and A3G proteins were reported to correspond well with reductions in the accumulation of viral reverse transcriptase (RT) products (34).

The different A3 family members were reported to inhibit the human non-LTR retrotransposons L1 and *Alu* with varying degrees of efficiency [reviewed in (23)]. The relatively high expression level of A3 proteins in human testis, ovary (A3G, A3F and A3C) and embryonic stem cells (A3B, A3C, A3D, A3F and A3G) points to a physiologically relevant role for these DNA deaminases in these cells in the protection from potentially deleterious effects caused by endogenous retroelement mobilization [reviewed in (23)]. Although both A3A and A3C include only one single CDA domain, A3A was demonstrated to be the most potent inhibitor of non-LTR retrotransposon mobilization. A3A restricted L1 and *Alu* retrotransposition frequencies by 85–99% and 75–98% (35), respectively, while A3C inhibited L1 and *Alu* by only 40–75% and 50–70%, respectively. There is no evidence for A3-mediated editing of members of the currently mobilized human-specific L1 subfamily L1Hs (36–38), and the mechanisms through which A3 proteins inhibit L1Hs retrotransposition are unclear to date. The CDA activity of many A3 proteins does not appear to be required since CDA mutants continue to inhibit L1 retrotransposition (36,39,40). Localization of the A3 proteins also does not appear to play a key role since both cytosolic and nuclear-localized A3 proteins effectively inhibit L1 retrotransposition (36,37,41).

Although there was no enhanced rate of G-to-A hypermutations detectable in L1 *de novo* insertions that occurred in the presence of A3A, mutating the catalytically active residues E72, C101 and C106 not only abolished the L1-inhibiting activity of A3A but even increased the L1 retrotransposition frequencies by 40 (E72A) to 70% (C101A/C106A) (37,42). It was hypothesized that the inactive A3A mutants relieve part of the L1 repression by blocking the binding of endogenous A3C and/or A3B proteins to L1 compounds (37). A3A can also restrict mobilization of the LTR-retrotransposon intracisternal A particle (IAP) (35) in cell culture assays, and it is active against the parvoviruses *Adeno-associated virus type 2* and *Minute virus of mouse* (43–45). Although A3A exerts its restricting effects on viral and retroviral targets primarily by mutating their genomes, and editing seems to be at the heart of many of these effects, A3A mutants devoid of detectable *in vitro* deaminase activity have been identified that can still restrict parvovirus (44). Also, in the presence of A3A, replicating viral genomes are decreased in *Adeno-associated virus type 2* producer cells (43), which mirrors the reduced levels of L1 reverse transcripts observed in A3A-expressing cells (46).

A3C is the most abundantly expressed of all the A3 genes across a wide range of tissues (17). All previously described A3C-mediated inhibitory effects such as the restriction of SIV_agm_Δ*vif* (47), HTLV-1 (48) and HIVΔ*vif* and HBV (49) are a consequence of cytidine deamination. The presence of G-to-A hypermutations in these viral genomes indicates that cytidine deamination plays a major role in the inhibition of these viruses. Whereas A3C is packaged into Δ*vif* HIV with a weak antiviral effect (50), A3C is a strong inhibitor of SIVΔ*vif* (47). A3C-encoded CDA activity can induce limited G-to-A mutations in HIV-1 that do not block viral replication, but rather contribute to viral diversity (51). Even though A3C, as well as A3A, A3B, A3F and mouse (m)A3, can inhibit mouse retroelements IAP and MusD, the involvement of editing is ambiguous (33). While A3C, A3G and A3F have been found to inhibit Ty1, concomitant G-to-A mutations have been found only in the case of A3G and A3F (52,53). A3C can cause extensive G-to-A mutations in the majority of the newly synthesized HBV DNA genomes but has little effect on HBV DNA synthesis (54,55). A3C can edit transfected *human papillomavirus* DNA and mitochondrial DNA (56,57). *Herpes simplex virus-1* is particularly vulnerable to the editing effects of A3C both in tissue culture and *in vivo* because it can impact both the titer and particle/PFU ratio (58).

We set out to elucidate the mechanism responsible for A3C-mediated inhibition of L1 retrotransposition by 40–75% (37,46,59,60) because A3C is the most abundantly expressed of all A3 genes across a wide range of tissues and cell types. We demonstrate that A3C-mediated L1 restriction is CDA independent and requires A3C dimerization. Two out of the three single mutations in the RNA-binding pocket caused a significant loss of L1 inhibition by A3C. A3C binds to L1 ORF1p in the presence of RNA, suggesting an interaction between A3C and L1 ribonucleoprotein particles (L1 RNPs). Furthermore, the amount of L1 cDNA synthesized by L1 RT is reduced by ∼50% if overexpressed A3C is present. Taken together, our data suggest that A3C dimers restrict L1 by means of a CDA-independent mechanism that is based on an RNA-dependent A3C/ORF1p interaction that inhibits the processivity of L1 RT.

## MATERIALS AND METHODS

### Plasmids

L1 retrotransposition reporter plasmids pJM101/L1_RP_ (61) and pDK101 (62) as well as the expression plasmids encoding C-terminally hemagglutinin (HA)-tagged A3A and A3C WT proteins and the A3A mutants E72A and C101A/C106A (37) have been described recently. Plasmids expressing HA-tagged A3C mutant proteins H66R, E68Q, C100S, K22A, F55A, W74A, R122A and N177A were generated by inserting the respective HindIII/XhoI fragments of the recently described pcDNA3.1(+)-based expression plasmids (63) into the pcDNA3.1/Zeo(+) multiple cloning site (Invitrogen) after HindIII/XhoI restriction.

### Cell culture, L1 retrotransposition reporter assays and statistical analyses

HeLa-JVM cells (64) were cultured in Dulbecco's modified Eagle's medium (DMEM) High Glucose (Biochrom AG) supplemented with 5% FCS (Biowest), 2 mM l-glutamine and 20 U/ml penicillin/streptomycin (Invitrogen). Osteosarcoma 143Btk- cells were grown in DMEM high glucose supplemented with 10% FCS, 2 mM l-glutamine, 20 U/ml penicillin/streptomycin and 1 mM nonessential amino acids. All cells were incubated in a humidified 5% CO_2_ incubator at 37°C and passaged using standard cell culture techniques. L1 retrotransposition frequencies were determined applying the rapid and quantitative transient L1 retrotransposition reporter assay described previously (37,65). Briefly, for each transfection, 2 × 10^5^ HeLa cells/well were seeded in six-well tissue culture dishes. The following day, each well was co-transfected with 0.5 μg of reporter plasmid pJM101/L1_RP_ and 0.5 μg of the respective mutant or A3 WT expression plasmid using 3 μl of FuGENE 6 transfection reagent (Roche Diagnostics) according to manufacturer’s instructions. For the titration experiment (Figure 4), 0.25–1.5 μg of the plasmid DNA encoding the R122A mutant or A3C-WT were each filled up to a total mass of 1.5 μg plasmid DNA using the empty vector pcDNA3.1/Zeo(+). Subsequently, each of the 1.5 μg-plasmid DNA samples was co-transfected with 0.5 μg of the pJM101/L1_RP_ reporter using 6 μl of FuGENE HD transfection reagent (Promega). Seventy-two hours after transfection, cells were selected for L1 retrotransposition events in 800 µg/ml G418 (Invitrogen) for 10–12 days. G418^R^ colonies were fixed and stained with Giemsa (Merck, Darmstadt, Germany) as described previously. Each co-transfection was performed once or twice in quadruplicate: In each case, three co-transfection experiments were used to quantify L1 retrotransposition activities in presence or absence of the respective WT or mutant A3 expression plasmid. The fourth co-transfection was used to isolate cell lysates to analyze expression of both L1 reporter element and WT or mutated A3 expression cassette. For each co-transfection that was performed in quadruplicate, transfection efficiency was monitored by co-transfecting 0.5 μg of plasmid phMGFP (expressing green fluorescent protein; Promega), 0.5 μg of pJM101/L1_RP_ and 0.5 μg of the respective mutant or WT A3 expression plasmid into 2 × 10^5^ HeLa cells seeded in parallel using FuGENE 6. EGFP-expressing cells were counted 24 h after transfection by flow cytometry. The percentage of green fluorescent cells was used to determine the transfection efficiency of each sample (66,67), which reproducibly ranged from 64 to 70%.

Comparisons of the effects of different WT and mutant A3 proteins on L1 retrotransposition frequencies were performed applying Student’s *t*-test with *P*-values adjusted for multiple comparisons according to the Bonferroni method. The statistical analysis was performed with SAS®/STAT software, version 9.3 SAS System for Windows. *P*-values, which were determined to measure the significance of the L1 inhibiting effect of each A3C mutant relative to the empty A3C expression plasmid (mock) or the A3C-WT protein, respectively, are listed in Supplementary Table S1.

### Toxicity assay

Toxicity assays were performed as previously described (35,37). Briefly, 2 × 10^5^ JM-HeLa cells were seeded per well of a six-well dish. The following day, cells were co-transfected with 0.5 µg of pcDNA3.1(+) (Invitrogen) expressing the neomycin resistance gene, and 0.5 µg of the A3C-WT or mutant expression plasmid or the empty vector pcDNA3.1/Zeo(+), respectively, using FuGene-HD transfection reagent (Promega) according to manufacturer’s instructions. Only in the case of the A3C mutant R122A, 1.5 µg of plasmid DNA were co-transfected with 0.5 µg of pcDNA3.1(+) to assure R122A protein levels comparable with the remaining A3C mutant proteins. Two days after transfection, G418 selection (800 µg/ml) was initiated and continued for 10 days. G418^r^ colonies were stained with Giemsa and counted. Each co-transfection experiment was performed in quadruplicate: The arithmetic mean of the number of G418^r^ colonies of three independent co-transfection experiments was determined and error bars were calculated. To also control for comparable expression levels of the different A3C-WT and mutant proteins, the fourth co-transfection was performed. Hence, one well of each co-transfection was used to isolate cell lysate 3 days after transfection.

### Immunoblot analysis

To analyze expression of WT and mutant A3 proteins, and of the tagged L1 reporter element, co-transfected HeLa cells were lysed 48 h after transfection using triple lysis buffer (20 mM Tris/HCl, pH 7.5; 150 mM NaCl; 10 mM EDTA; 0.1% sodium dodecyl sulphate (SDS); 1% Triton X-100; 1% deoxycholate; 1× complete protease inhibitor cocktail [Roche]), and lysates were cleared by centrifugation. Twenty micrograms of each protein lysate were boiled in Laemmli buffer, loaded on 12% polyacrylamide gels, subjected to SDS-polyacrylamide gel electrophoresis (PAGE) and electroblotted onto nitrocellulose membranes. After protein transfer, membranes were blocked for 2 h at room temperature in a 10% solution of nonfat milk powder in 1× PBS-T [137 mM NaCl, 3 mM KCl, 16.5 mM Na_2_HPO_4_, 1.5 mM KH_2_PO_4_, 0.05% Tween 20 (Sigma-Aldrich Chemie GmbH)], washed in 1× PBS-T and incubated overnight with the respective primary antibody at 4°C. HA-tagged A3 proteins and L1 ORF1p were detected using an anti-HA antibody (Cat.# MMS-101P; Covance Inc.) in a 1:5000 dilution and the polyclonal rabbit-anti-L1 ORF1p antibody #984 (68) in a 1:2000 dilution, respectively, in 1× PBS-T containing 5% milk powder. Subsequently, membranes were washed thrice in 1× PBS-T. As secondary antibodies to detect HA-tagged A3 proteins and L1 ORF1p, we used horseradish peroxidase (HRP)-conjugated anti-mouse IgG antibody at a dilution of 1:7500, and HRP-conjugated anti-rabbit IgG antibody (Amersham Biosciences Europe GmbH) at a dilution of 1:30 000, respectively, in 1× PBS-T/1.67% milk powder and incubated the membrane for 2 h. Subsequently, the membrane was washed thrice for 10 min in 1× PBS-T. ß-actin expression was detected using a monoclonal anti-ß-actin antibody (clone AC-74, Sigma-Aldrich Chemie GmbH) at a dilution of 1:30 000 as primary antibody, and an anti-mouse HRP-linked species-specific antibody (from sheep) at a dilution of 1:10 000 as secondary antibody. For the detection of endogenous S6 ribosomal protein in sucrose gradient fractions, blots were probed with anti S6 ribosomal protein rabbit mAb (5G10; 1:10^3^ dilution in 5% bovine serum albumin dissolved in TBST; Cell Signaling Technology), for 1 h at room temperature. Anti-rabbit HRP (1:10^4^ dilution, GE Healthcare) was used as secondary antibody. Immunocomplexes were visualized using lumino-based ECL immunoblot reagent (Amersham Biosciences Europe GmbH).

### Immunofluorescence microscopy

For immunofluorescence studies in HeLa cells and 143Btk- cells, 10^5^ cells/well were seeded on coverslips in 12-well cell culture dishes. Next day, cells of each well were transfected with 3.3 µg of plasmid DNA applying FuGENE HD (Promega) according to manufacturer’s instructions. Three days after transfection, cells were washed once with Dulbecco’s phosphate-buffered saline (Biochrom AG). Cells were fixed in 4% paraformaldehyde in phosphate buffered saline (PBS) for 15 min at room temperature, washed in PBS and permeabilized in 1% Triton-X-100/PBS for 10 min. Subsequently, cells were washed thrice for 2 min in PBS, blocked in 0.1% PBS-Triton X-100/5% Albumin Fraction V for 30 min at room temperature and finally incubated with primary antibodies in 5% Albumin Fraction V-PBS for 1 h at room temperature.

For the staining of L1 ORF1p-T7 expressed from pDK101, cells were incubated with an anti-T7 antibody (Abcam) at a dilution of 1:250. Subsequently, cells were washed thrice for 5 min with PBS. To stain for the expression of HA-tagged A3C-WT and mutant proteins, cells were incubated with an fluorescein isothiocyanate-labeled anti-HA antibody (GenScript) in a 1:500 dilution together with the secondary antibody for L1 ORF1p-T7 detection in 5% Albumin Fraction V-PBS for 30 min and washed thrice for 5 min in PBS. The secondary antibody used for the detection of L1 ORF1p-T7 was an anti-rabbit Alexa647 antibody (Invitrogen/Life Technologies) at a 1:1000 dilution. Nuclei were stained with DAPI (1 µg/ml) for 1 min, followed by washing three times for 10 min in PBS. Coverslips were mounted in Fluoromount-G (SouthernBiotech). Finally, cellular protein expression was analyzed by confocal microscopy. Images were acquired on a fully automated Axio-ObserverZ1 microscope equipped with an ApoTome optical sectioning unit (Carl Zeiss, Jena, Germany). From each culture grown on n coverslips (n = 3 for each co-expressed A3C mutant; n = 5 for co-expressed A3C-WT), one high-resolution image per coverslip culture was assembled by acquisition of 25 adjacent fields of view per coverslip culture using the MosaiX module (Carl Zeiss). Morphometric analysis for A3C-WT (or mutant)/ORF1p-co-localization as well as nuclei quantification was performed using the CellProfiler Cell Image Analysis Software [www.cellprofiler.org; Broad Institute, Cambridge, MA, USA; (69)] and post-processed with the help of APOCELL (https://pypi.python.org/pypi/apocell).

To analyze for potential co-localization of L1 ORF1p-T7 or A3C-HA fusion proteins with the stress granule marker G3BP, the following primary and secondary antibodies and dilutions were applied: anti-T7 antibody (1:500 dilution; Novagen/Merck AG, Darmstadt, Germany), anti-HA antibody (1:250 dilution; Novus Biologicals). As secondary antibodies, anti-mouse Alexa488 (1:500 dilution; Molecular Probes) or anti-mouse Alexa594 (1:500 dilution; Invitrogen) and anti-goat Alexa546 (1:500 dilution; Molecular Probes) were used, respectively. To detect the stress granule marker G3BP, anti-G3BP antibody (1:250 dilution; Sigma-Aldrich) and secondary antibody anti-rabbit Alexa488 (1:500 dilution; Molecular Probes) were used.

To evaluate whether the P-body–specific marker rck/p54 co-localizes with L1 ORF1p-T7 or A3C-HA fusion proteins, the following antibodies and dilutions were used: anti-T7 antibody (1:500; Novagen; anti-mouse Alexa488/1:500; Molecular Probes), anti-HA (1:250; Novus Biologicals; anti-goat Alexa546/1:500; Molecular Probes) and anti-rck/p54 (1:100; MBL International; anti-rabbit Alexa647/1:500; Molecular Probes).

### Velocity sucrose gradient fractionation

To generate cell lysates to be fractionated by velocity sucrose gradient centrifugation, 2 × 10^6^ HeLa cells per co-transfection were seeded in a T-75 cell culture flask. Twenty-four hours later, cells were co-transfected with 10 µg of the L1 reporter plasmid pJM101/L1_RP_ and 10 µg of the A3C-WT or mutant expression plasmid or the empty expression vector pcDNA 3.1/Zeo(+), respectively, using FuGENE HD. Three days later, cells were washed twice with cold PBS, detached with Trypsin EDTA pelleted for 5 min at 3000*g* and 4°C and incubated on ice for 5 min in lysis buffer [1.5 mM KCl, 2.5 mM MgCl_2,_ 20 mM Tris–HCl, pH 7.4, 1% deoxycholate, 1% Triton X-100 and 1× complete EDTA-free protease inhibitor cocktail (Roche) (62)]. The lysates were cleared by centrifugation in a benchtop centrifuge at 162*g* for 10 min followed by 18 000*g* for 30 s. Subsequent sucrose gradient centrifugation was performed as described by Huthoff *et al.* (70). Briefly, cell lysates (110 µl each) were loaded on top of a 10–15–20–30–50% sucrose step gradient in a buffer [80 mM NaCl, 5 mM MgCl_2_, 20 mM Tris–HCl, pH 7.4, 1 mM DTT, 1× complete EDTA-free Protease Inhibitor Cocktail (Roche) (62)] in ultra-clear centrifuge tubes (13 × 51 mm, Beckman Coulter) and centrifuged for 2 h at 39 000*g* at 4°C in an MLS-50 rotor (Beckman Coulter). After centrifugation, the samples were sequentially removed from the top of the gradient as 12 different fractions (84 µl per fraction). Sucrose concentrations of the different fractions were measured using a refractometer (AFAB Enterprises). Thirty microliters of each fraction were loaded per lane on a protein gel, separated by SDS-PAGE and subjected to immunoblot analysis.

### Isolation of L1 RNPs

To isolate L1 RNPs, HeLa cells were plated at 6 × 10^6^ cells per T-175 cell culture flask. Cells were transfected 24 h later with 20 µg L1 reporter plasmid pDK101 (71) per flask using FuGENE 6 or FuGENE HD transfection reagent. Three days after transfection, medium was replaced by DMEM-complete supplemented with 200 µg/ml hygromycin B and exchanged daily until day 7 after transfection. When hygromycin selection was complete, selection medium was replaced by DMEM-complete, and each T-175 culture was transfected with 20 µg of A3 expression plasmid using FuGENE 6. On the same day, one T-175 flask was seeded with 6 × 10^6^ untransfected HeLa cells to be used as negative control. Three days later, transfected and untransfected cells were harvested and whole-cell lysates were prepared as described recently (71). Preparation of the sucrose cushion and ultracentrifugation were performed as reported previously (71). As additional negative controls, one sucrose cushion was layered only with lysis buffer or with H_2_O, respectively, and subjected to the same procedures.

### Co-immunoprecipitation

For co-immunoprecipitation, 1.6 × 10^6^ HeLa cells were seeded in T-75 cell culture flasks 1 day before transfection using 8 µg of L1 reporter plasmid and FuGENE 6 according to the manufacturer’s protocol. Three days later, cells were subjected to hygromycin B selection (0.2 mg/ml). After 4 days of selection, cells were transfected with 8 µg of the A3 expression plasmid using FuGENE 6 or FuGENE HD transfection reagent. Three days later, cells were washed twice with cold phosphate-buffered saline/5% PMSF (PBSP), detached with a cell scraper in 10 ml PBSP and pelleted for 5 min at 3000*g* and 4°C. Cells were incubated on ice for 5 min in lysis buffer [25 mM Tris–HCl, pH 7.5, 150 mM NaCl, 5 mM MgCl_2_, 5% Glycerol, 1% IGEPAL CA-630, 1 mM DTT, 1× complete protease inhibitor cocktail (Roche)]. To precipitate HA-tagged A3 proteins, the cleared lysates were incubated with 50 µl of anti-HA Affinity Matrix Beads (Roche) overnight or 1 h at 4°C under gentle agitation. For RNase treatment, the cleared lysate was incubated with 50 µl of anti-HA Affinity Matrix Beads and RNase A (Qiagen) at a final concentration of 50 µg/ml. The beads were washed five times with ice-cold lysis buffer. Subsequently, the pelleted beads were boiled in SDS loading buffer for 8 min at 95°C before separation by SDS-PAGE.

### RNA isolation, reverse transcriptase-polymerase chain reaction and L1 element amplification protocol reaction

RNA isolation, reverse transcriptase-polymerase chain reaction (RT-PCR) and L1 element amplification protocol (LEAP) reaction procedures were adopted from (71) with the following modifications: A 50 µl of aliquot of each pelleted sample was used to isolate RNA using TRIzol reagent (Invitrogen) according to the manufacturer’s protocol. The RNA was resuspended in 20 µl of DEPC-treated water and quantified applying standard spectrophotometric methods. RNA (0.25 µg) in DEPC-treated water (8 µl) was treated with DNase I (Promega), incubated at 65°C for 10 min and chilled on ice. Next, RNA was reverse transcribed using SuperScript III RT (Invitrogen) and 0.8 mM LEAP primer (5′-GAGCACAGAATTAATACGACTCACTATAGGTTTTTTTTTTTTVN-3′) according to the manufacturer’s instructions in a final volume of 20 µl. As a negative control for the RT reaction, RNA was omitted from one RT reaction (Figure 8C, no RNP/RNA). Subsequently, 0.5 µl of each RT reaction were used in PCR reactions applying primers LINKER (5′-GCGAGCACAGAATTAATACGACT-3′) and ‘L1 3′ end’ [5′-GGGTTCGAAATCGATAAGCTTGGATCCAGAC-3′ (71)]. Thirty microliters of PCR products were run per lane on a 2% agarose gel.

The LEAP reaction was performed as described previously (71) with the following modifications: a 1-µl aliquot of each pelleted sample was incubated with 50 mM Tris–HCl (pH 7.5), 50 mM KCl, 5 mM MgCl_2_, 10 mM DTT, 0.4 mM LEAP primer, 20 U RNaseOut (Invitrogen), 0.2 mM dNTPs and 0.05% Tween 20 in a final volume of 50 µl for 1 h at 37°C. Reactions with RNPs from untransfected HeLa cells (HeLa) or in the absence of RNPs (No RNP/RNA) were used as negative controls. After incubation, 1 µl of each LEAP reaction was used in a standard 30 µl of PCR using AmpliTaq DNA polymerase (Applied Biosystems) and 0.4 mM of each linker PCR primer and L1.3 end primer (71) according to the manufacturer’s protocol. As ‘No template’ control, LEAP cDNA was omitted from one reaction (No template). PCR conditions were the following: one cycle 94°C for 3 min, 35 cycles 94°C for 30 s at 56°C, one cycle 10 min at 72°C. The complete reaction was visualized on a 2% agarose gel. Quantification of the LEAP products was performed by applying the program Bio-1D Version 12.11 (Vilber Lourmat, Marne-la-Vallée, France).

## RESULTS

### Deaminase-deficient A3C retains its L1-restricting effect

**Figure 1.**
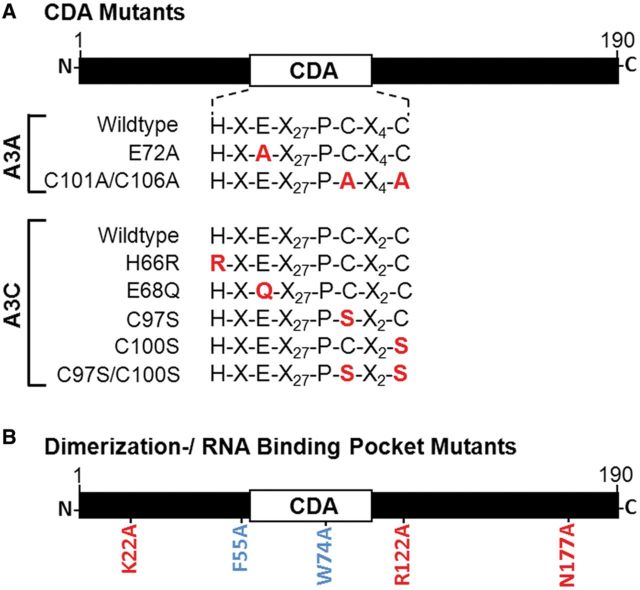
Schematic representation of A3A and A3C mutant constructs used in this study. (**A**) CDA mutants. A3A and A3C include the CDA motif HXEX_27_PCX_2_C. Point mutations were introduced into the A3A- and A3C-encoding DNAs resulting in mutations in the putative CDA domains. (**B**) Dimerization domain and RNA-binding pocket mutants of A3C. Blue font, mutations F55A and W74A that are located in the dimerization domain; Red font, mutations K22A, R122A and N177A that are located in the RNA-binding pocket domain. To evaluate whether A3C-WT and/or the mutant derivatives used in this study have any off target effects that might impact cell viability, and, as a consequence, hamper the identification of potential direct effects of any mutant proteins on L1 retrotransposition, we conducted toxicity assays in HeLa cells (37) (Supplementary Figure S1). Results indicate that none of the overexpressed WT or mutant A3C proteins has any considerable effect on cell viability.

To elucidate the mechanism that is responsible for the restriction of L1 retrotransposition by A3C [reviewed in (23)], we first investigated whether an intact CDA domain was required for A3C-mediated L1 inhibition. To answer the question of whether the presumed catalytic active site of A3C (H-X-E-X_27-_P-C-X_2-_C) is involved in the inhibition of L1 retrotransposition, we introduced mutations that were reported to abolish the editing activity in the context of A3G (72) and the L1-inhibiting effect of A3A (37) (Figure 1A).

**Figure 2.**
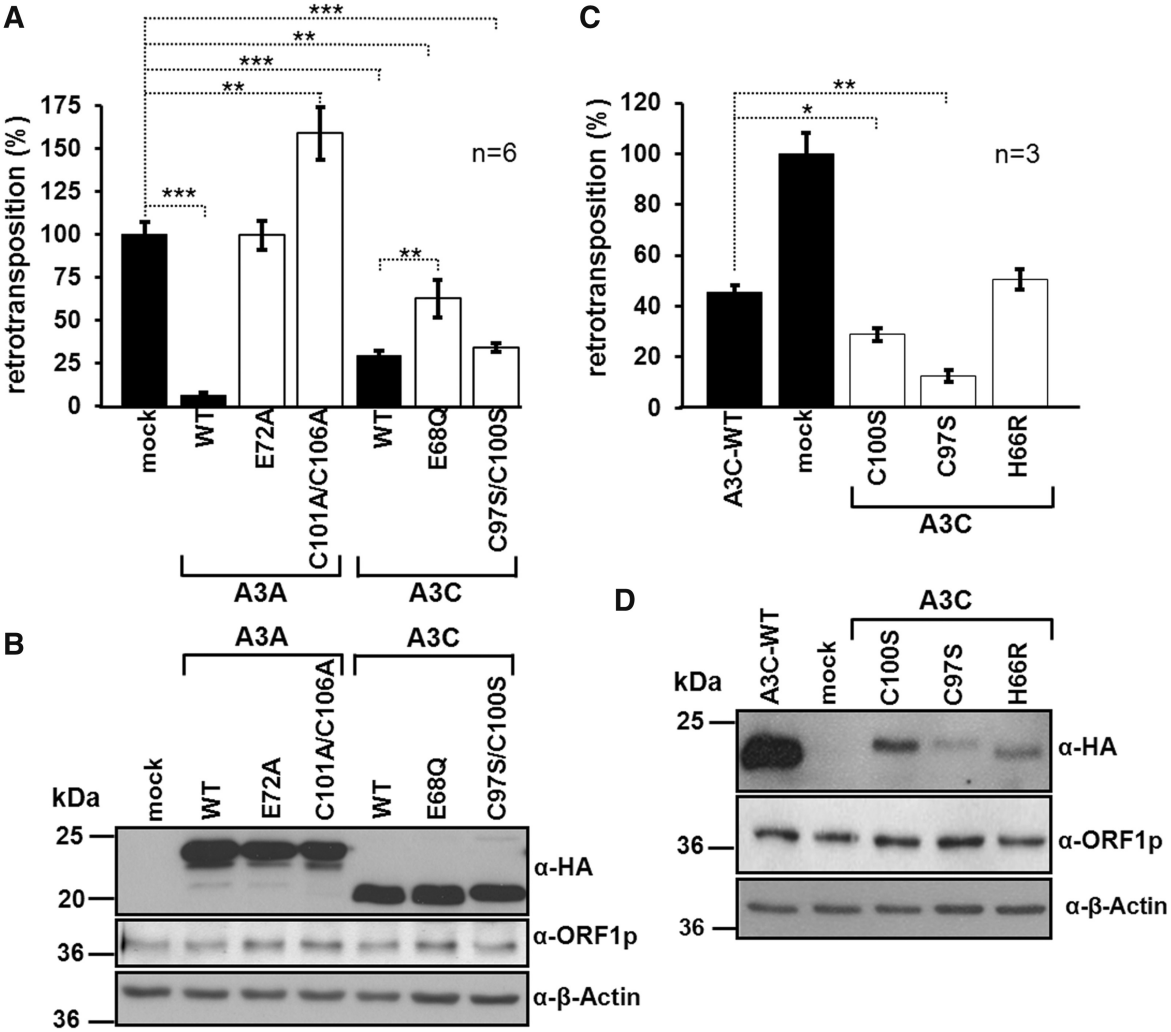
A3C does not require an intact CDA domain for L1 restriction (**A**) Relative L1 retrotransposition frequencies in the presence of A3A, A3C and their corresponding CDA single and double mutants. HeLa cells were co-transfected with 0.5 µg of both L1 reporter plasmid pJM101/L1_RP_ and WT or mutant A3 expression plasmids. The number of retrotransposition events in the presence of the empty expression plasmid pcDNA3.1/Zeo+ (mock) was set as 100%. Each co-transfection experiment and subsequent retrotransposition reporter assay was carried out twice in triplicates. Each bar depicts the arithmetic mean ± SD of the relative retrotransposition frequencies obtained from six individual co-transfection experiments (*n* = 6). Absolute retrotransposition frequencies and *P*-values (*P < 0.05, **P < 0.01, ***P < 0.001) are listed in Supplementary Table S1. (**B**) Immunoblot analysis to control for comparable expression levels of WT and mutant A3A and A3C proteins. Each lane was loaded with 20 µg of cell lysate isolated from HeLa cells 48 h after co-transfection of A3 expression plasmid and pJM101/L1_RP_. Expression of HA-tagged A3 proteins and L1 ORF1p was detected with anti-HA and anti-ORF1p antibodies, respectively. β-actin (∼42 kDa) expression served as loading control. (**C**) Effects of A3C-CDA domain mutants C97S, C100S and H66R on L1 retrotransposition frequencies. HeLa cells were co-transfected with 0.5 µg of both L1 reporter plasmid pJM101/L1_RP_ and WT or mutant A3C expression plasmids. The number of retrotransposition events in the presence of the empty expression plasmid (mock) was set as 100%. Each co-transfection experiment, and subsequent retrotransposition reporter assay, was performed thrice (no replicates). Each bar depicts the arithmetic mean ± SD of the relative retrotransposition frequencies obtained from three individual co-transfection experiments (*n* = 3). Absolute retrotransposition frequencies and *P*-values are listed in Supplementary Table S1. (**D**) Immunoblot analysis of L1 ORF1p, A3C-WT, C97S, C100S and H66R mutant expression after co-transfection of the L1 retrotransposition reporter plasmid and WT or mutant A3C expression plasmids. Twenty micrograms of whole-cell extract were loaded per lane. Expression of HA-tagged A3C proteins and L1 ORF1p was detected with anti-HA and anti-ORF1p antibodies, respectively. β-actin expression served as loading control.

A3G active-site mutants H257R, E259Q, C288S and C291S exhibit CDA activities that are decreased by 90–95% relative to A3G-WT (73). Mutating the corresponding A3A active-site residues H70, E72 and C106 to E72Q, H70R and C106S abolishes deaminase activity and the L1-restricting effect of A3A (43,44). Thus, we mutated the corresponding residues H66, E68 and C100 in A3C (Figure 1A) to H66R, E68Q and C100S, respectively, expecting elimination of A3C CDA activity. Simultaneously mutating the Zn^2+^-coordinating Cys residues at position 97 and 100 to Ser (C97S/C100S) did not affect A3C-mediated L1 restriction significantly (*P* = 0.328, Figure 2A and Supplementary Table S1), and mutating both Cys residues separately even slightly increased the L1 inhibition from 54 to 87% (*P* = 0.0023) or 71% (*P* = 0.0349), respectively (Figure 2C and Supplementary Table S1). These findings clearly provide evidence that deaminase activity is not required for A3C-mediated L1 restriction. Replacing Glu68 by Gln (E68Q) still resulted in a significant inhibition of L1 by ∼40% (*P* = 0.0044, Figure 2A). In contrast, mutating the same Cys and Glu residues in the CDA domain of A3A (C101A/C106A, E72A) not only abrogated A3A-mediated L1 restriction but even increased the number of detectable retrotransposition events by 60–70% (C101A/C106A; Figure 2A), as reported previously (37). Replacing His66 by Arg led to the A3C CDA mutant H66R, and made no significant difference (Figure 2C) to the L1-inhibiting properties of A3C (*P* = 1.0). Since our *Escherichia coli* mutation assays demonstrate that the A3C CDA mutants H66R, E68Q, C97S, C100S and C97S/C100S are devoid of any meaningful DNA-editing activity relative to A3C-WT (Supplementary Figure S2), the retrotransposition reporter assays (Figure 2) show that, in contrast to A3A, A3C does not require an enzymatically active CDA domain to restrict L1 retrotransposition.

To exclude the possibility that the observed differences in retrotransposition frequencies can be attributed to varying L1 and/or A3 protein levels, we assessed L1 ORF1p and A3 expression in a parallel set of co-transfected HeLa cell cultures (Figure 2B and D). Immunoblot analysis of cell extracts isolated 48 h after transfection showed that comparable amounts of L1 ORF1p as well as mutant and WT A3A and A3C proteins were expressed in the experiments comparing the different effects of corresponding A3A and A3C CDA mutants with each other (Figure 2B). Although the expression level of A3C-WT exceeded those of the A3C mutants C100S, C97S and H66R (Figure 2D), the mutants were still inhibiting L1 by 50–87% (Figure 2C), emphasizing that an intact CDA domain is not required for A3C-mediated L1 restriction.

### A3C dimerization is required for L1 restriction

**Figure 3.**
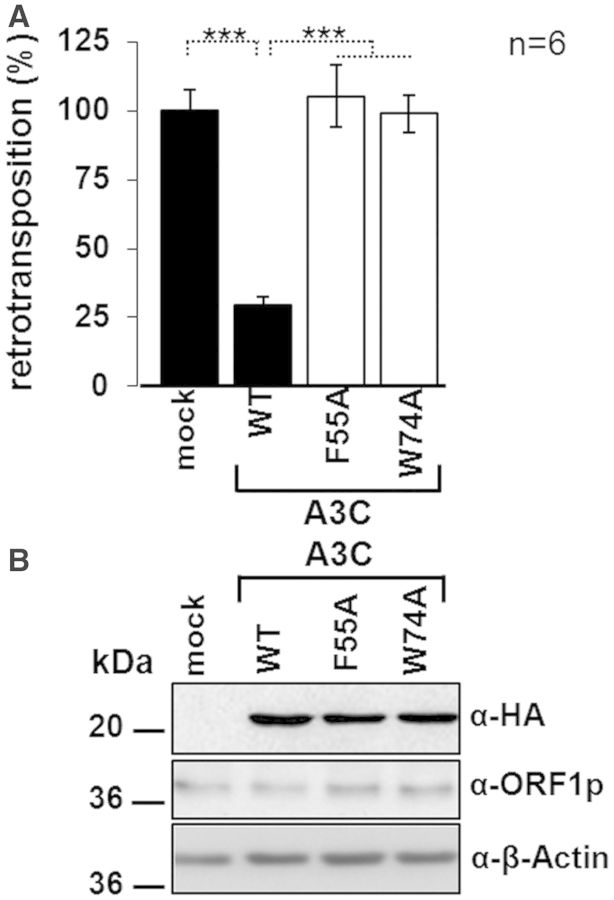
A3C dimerization is required for L1 restriction by A3C. (**A**) L1 retrotransposition reporter assays in the presence of A3C dimerization mutant proteins F55A and W74A. The L1 reporter plasmid pJM101/L1_RP_ was co-transfected with expression plasmids coding for A3C-WT or its mutants F55A and W74A. G418^R^ selection for retrotransposition events followed 1 day after transfection. L1 retrotransposition frequencies were determined by counting G418^R^ HeLa colonies. Each co-transfection experiment, and subsequent retrotransposition reporter assay, was carried out twice in triplicates. Relative retrotransposition frequencies are indicated as bar diagram. The number of retrotransposition events obtained after co-transfection of pJM101/L1_RP_ with the empty expression plasmid (mock) was set as 100%. Each bar depicts the arithmetic mean ± SD of the relative retrotransposition frequencies obtained from six individual co-transfection experiments (*n* = 6). Absolute retrotransposition frequencies and *P*-values (*P < 0.05, **P < 0.01, ***P < 0.001) are listed in Supplementary Table S1. (**B**) Immunoblot analysis of A3C and L1 ORF1p expression in HeLa cells after co-transfection of the L1 reporter plasmid with the respective A3C-WT and mutant A3C expression construct (F55A, W47A). Whole-cell lysates were prepared 2 days after co-transfection and subjected to immunoblot analysis using antibodies against L1 ORF1p (α-ORF1p) or HA-tag (α-HA). An amount of 20 µg of whole-cell extract was loaded per lane. β-actin protein levels were analyzed as loading controls.

Next, we wanted to investigate whether L1-restricting A3C activity requires protein multimerization because oligomerization is essential for the inhibition of SIVΔ*vif* replication (63). For that purpose, we chose the dimerization-deficient A3C mutants F55A and W74A (Figure 1B) because it was demonstrated that amino acid residues F55 and W74 are essential for dimerization and/or oligomerization of A3C, respectively (Supplementary Figure S3). Mutating these residues was also reported to abolish the capability of A3C to introduce G-to-A hypermutations in an *E. coli* mutation assay (63). To determine whether L1 inhibition requires oligomerization of A3C, expression plasmids encoding A3C-WT and mutant proteins F55A and W74A were separately co-transfected with the L1 retrotransposition reporter plasmid pJM101/L1_RP_, and the effect of the mutant proteins on L1 retrotransposition was analyzed (Figure 3A). We found that in contrast to the A3C-WT protein, dimerization-deficient mutants F55A and W74A (63) were unable to restrict L1 retrotransposition (Figure 3A and Supplementary Table S1; F55A versus Mock: *P* = 0.1; W74A versus Mock: *P* = 0.1), indicating that A3C oligomerization is required for A3C-mediated L1 restriction. To evaluate the possibility that the numbers of L1 retrotransposition events in A3C-WT and F55A or W74A-transfected cells is a consequence of discrepancies in WT and mutant A3C protein levels, we assessed expression levels of WT and mutant A3C proteins in a parallel set of co-transfected HeLa cells. Immunoblot analysis of cell extracts isolated 2 days after co-transfection showed that comparable amounts of A3C proteins were expressed (Figure 3B).

### R122A mutation in the putative RNA-binding pocket of A3C abrogates L1 inhibition by A3C

**Figure 4.**
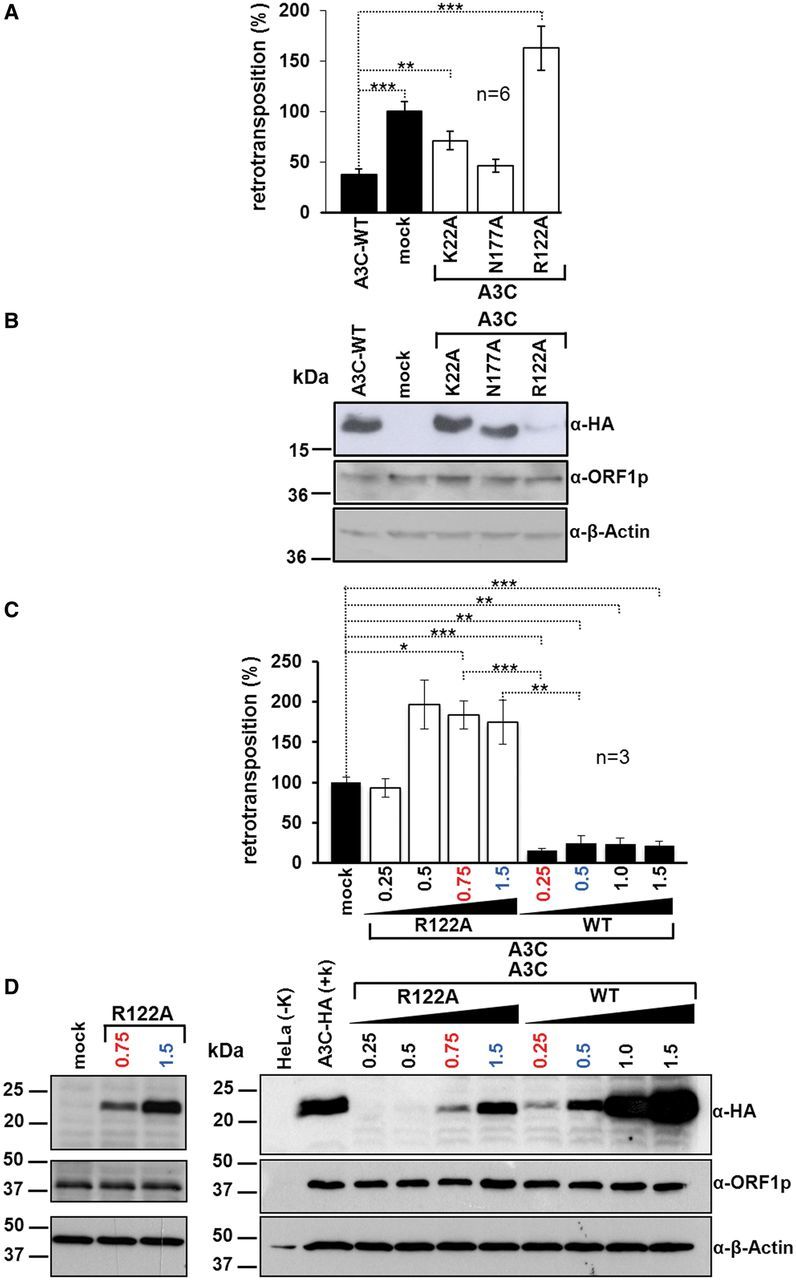
Effects of mutations in the putative RNA-binding pocket of A3C on L1 restriction. (**A**) Relative L1 retrotransposition frequencies in the presence of ectopically expressed A3C-WT and the mutant proteins K22A, N177A and R122A. HeLa cells were co-transfected with 0.5 µg of both L1 reporter pJM101/L1_RP_ and WT or mutant A3C expression plasmids. After 11 days of G418 selection, the number of G418^R^ colonies was determined. The number of retrotransposition events in the presence of the empty A3C expression plasmid (mock) was set as 100%. Each co-transfection experiment, and subsequent retrotransposition reporter assay, was carried out twice in triplicates. Each bar depicts the arithmetic mean ± SD of the relative retrotransposition frequencies obtained from six individual co-transfection experiments (*n* = 6). Absolute retrotransposition frequencies and *P*-values (*P < 0.05, **P < 0.01, ***P < 0.001) are listed in Supplementary Table S1. (**B**) Immunoblot analysis of the expression of A3C-WT and the mutants K22A, N177A and R122A after co-transfection with the L1 reporter pJM101/L1_RP_. Expression of the L1 reporter and A3C proteins was detected using antibodies against L1 ORF1p (α-ORF1p) or HA-tag (α-HA), respectively. An amount of 20 µg of whole-cell extracts was loaded per lane. β-actin protein levels were analyzed as loading controls. (**C**) Titration of A3C-WT and A3C-mutant R122A protein levels against L1 retrotransposition. HeLa cells were co-transfected with 0.5 µg of pJM101/L1_RP_ and variable amounts of A3C-WT (0.25, 0.5, 1 or 1.5 µg) of A3C-R122A mutant-encoding plasmid DNA (0.25, 0.5, 0.75 or 1.5 µg). The total amount of co-transfected plasmid DNA was maintained at 2 µg by adding empty parental A3C expression vector pcDNA3.1/Zeo(+). Following 11 days of G418 selection, the number of G418^R^ colonies was determined. The number of retrotransposition events in the presence of the empty expression plasmid (mock) was set as 100%. Data represent arithmetic means ± SD of three independent experiments. Absolute retrotransposition frequencies and *P*-values are listed in Supplementary Table S1. (**D**) Immunoblot analysis of A3C-WT and R122A protein expression during titration. Cell lysates were isolated 3 days after co-transfection of pJM101/L1_RP_ and 0.25–1.5 µg of A3C-WT or the R122A mutant expression plasmid. Expression of the L1 reporter and A3C proteins was detected using antibodies against L1 ORF1p (α-ORF1p) or HA-tag (α-HA), respectively. An amount of 20 µg of whole-cell extracts was loaded per lane. β-actin protein levels were analyzed as loading controls. Comparable amounts of A3C-WT and R122A mutant proteins were observed after transfection of 0.25 µg of A3C-WT and 0.75 µg of R122A expression construct (numbers in red) or after transfection of 0.5 µg of A3C-WT and 1.5 µg of R122A expression construct (numbers in blue), respectively.

By using a structure-based algorithm for automated pocket extraction, a putative RNA-binding pocket was recently located distal from the Zn^2+^-coordinating deaminase motif of A3C (63). Since evidence for binding of 5.8S ribosomal RNA (rRNA) and 7SL RNA to this substrate-binding pocket has been provided (63), we evaluated whether it plays a role in A3C-mediated L1 inhibition. We tested the same mutant A3C proteins—i.e. K22A, R122A and N177A—that contain mutations near the RNA-binding pocket (Figure 1B) and were analyzed for their inhibitory effect on WT and SIVΔ*vif* replication recently (63). We found that A3C-WT and its N177A mutant inhibited L1 retrotransposition similarly by ∼62 and 54%, respectively. The N177A mutation did not affect A3C-mediated L1 restriction significantly (*P* = 0.1). However, the K22A mutation reduced the L1 restricting effect to only ∼29% relative to mock-transfected cells (*P* = 0.0015, Figure 4A and Supplementary Table S1). Initial retrotransposition reporter assays also suggested that the R122A mutation in A3C not only fully restored the retrotransposition activity of the L1 reporter to 100% (defined as the number of L1 retrotransposition events in the absence of overexpressed A3C in HeLa cells) (Figure 4A), but actually significantly increased retrotransposition frequency by ∼65% relative to the empty expression vector (*P* = 0.0328, Figure 4A). These data suggest that the RNA-binding pocket domain of A3C plays a role in L1 inhibition. However, immunoblot analysis of cell extracts isolated from HeLa cells that were co-transfected with the L1 reporter plasmid pJM101/L1_RP_ and WT or mutant A3C expression constructs, revealed poor R122A expression levels (Figure 4B), which may at least in part account for the absence of any L1-restricting effect of R122A.

To evaluate whether, with equal amounts of actual protein expressed, L1 retrotransposition is restored in the presence of the R122A mutant, we titrated A3C-WT and R122A mutant protein levels against the resulting L1 reporter retrotransposition activities in HeLa cells (Figure 4C and Supplementary Table S1). Expression of comparable amounts of R122A and A3C-WT protein (0.75 µg of R122A plasmid DNA versus 0.25 µg of A3C-WT plasmid DNA; Figure 4D) led to a significant ∼1.8-fold increase (*P* = 0.0151) and an ∼7-fold decrease (*P* = 0.0003) of L1 retrotransposition, respectively (Figure 4C). Increasing the expression of comparable amounts of both proteins by transfecting 1.5 µg of R122A plasmid DNA and 0.5 µg of A3C-WT plasmid DNA resulted in similar effects: R122A enhanced L1 retrotransposition by ∼1.7-fold (*P* = 0.1022), whereas A3C-WT inhibited L1 by ∼4-fold (*P* = 0.0035). These data confirmed that the restoration of the relative L1 retrotransposition frequency of 100%, observed after co-expression of the R122A mutant (Figure 4A and C), is not the consequence of minor R122A expression levels (Figure 4B and D), but is due to the R122A mutation that affects the putative RNA-binding pocket. There were no significant differences between L1-enhancing effects resulting from the transfection of increasing amounts (0.5, 0.75 and 1.5 µg) of R122A plasmid DNA (*P* = 0.5866) nor between L1-restricting effects of increasing A3C-WT protein levels resulting from transfection of 0.25, 0.5, 1 or 1.5µg A3C-WT plasmid DNA (*P* = 0.4190). The 1.7- to 2-fold increase of L1 retrotransposition resulting from co-transfection of 0.5–1.5 µg of the R122A expression construct (Figure 4C and D) suggests that R122A mutant proteins may relieve part of the L1 repression caused by endogenously expressed A3C-WT in HeLa cells by forming dimers with A3C-WT (37,74) and thus blocking their L1-inhibiting effects. Interestingly, 0.25 µg of the R122A-expressing plasmid only restored L1 retrotransposition frequency but did not enhance L1 mobilization relative to mock-transfected cells (*P* = 0.1, Figure 4C and D), suggesting that the R122A level in the co-transfected cells is too low to affect the L1-inhibiting effect of endogenously expressed A3C-WT.

Taken together, we could show that the L1 restricting effect of the RNA-binding pocket mutant N177A is only slightly attenuated relative to A3C-WT, while the K22A mutant protein reduced L1 retrotransposition frequency by only ∼29%. The R122A mutation not only abolished the L1-inhibiting effect of A3C-WT entirely, but also even increased L1 retrotransposition frequencies by 62–96% (Figure 4). These data suggest that the ability to bind RNA is crucial for the L1-restricting activity of A3C.

### Ectopic expression of A3C causes a shift of L1 ORF1p to sucrose gradient fractions of lower molecular mass

**Figure 5.**
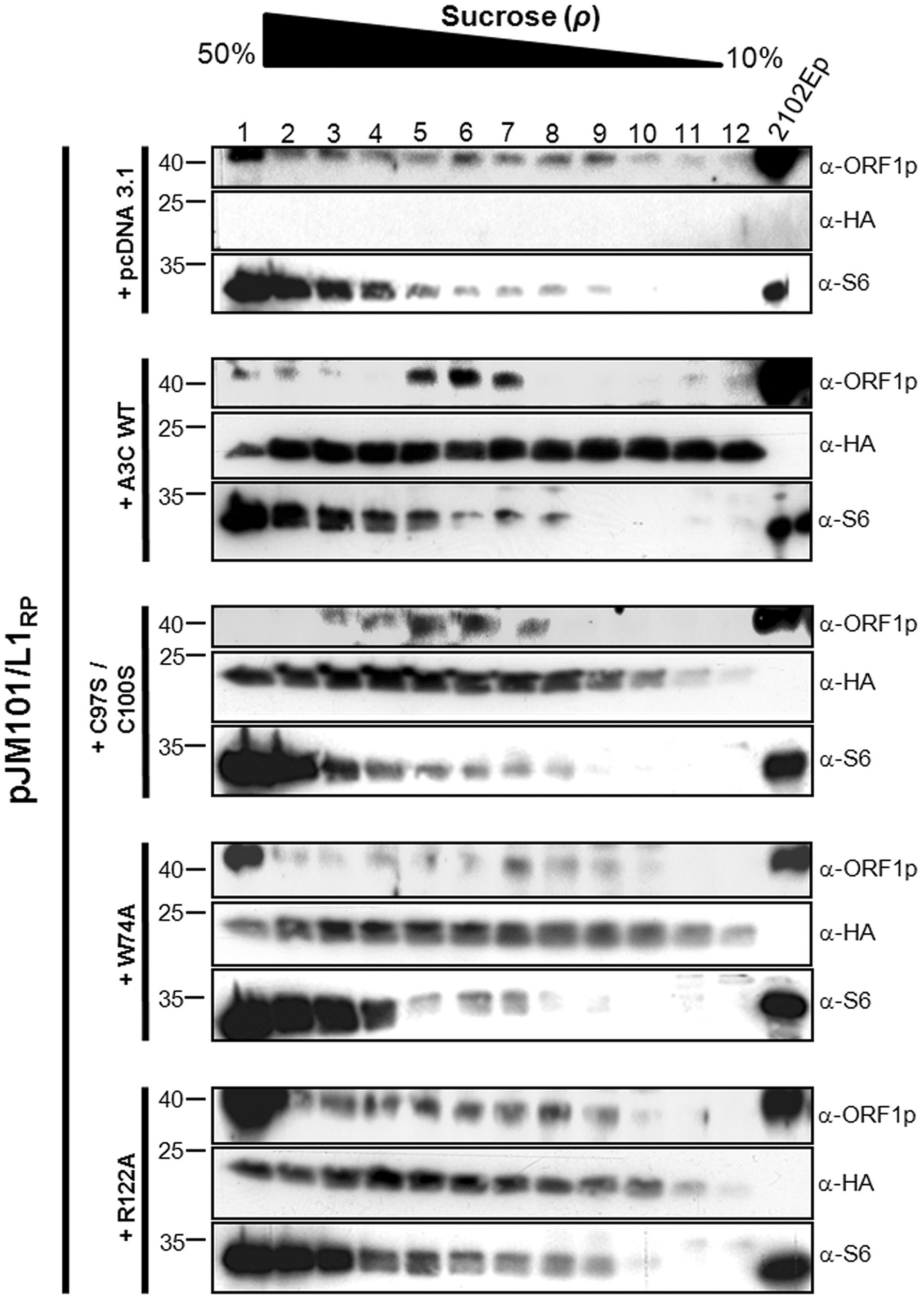
Velocity sucrose gradient fractionation of A3C-WT or mutant proteins and L1 ORF1p containing complexes. Whole-cell lysates from HeLa cells co-transfected with plasmids expressing WT or mutant A3C proteins and L1 reporter plasmid pJM101/L1_RP_ were layered over 10–50% sucrose containing Mg^2+^. The lowest number fraction corresponds to the bottom of the gradient. ORF1p (40 kDa), A3C WT or mutant (24 kDa) and S6 ribosomal marker proteins (32 kDa) were detected by immunoblot analysis using anti-ORF1p, anti-HA and anti-S6 antibodies, respectively. In the absence of ectopically expressed A3C proteins (pcDNA 3.1) and in the presence of overexpressed W74A or R122A mutant proteins, ORF1p co-localizes predominantly with ribosomes and polyribosomes in the bottom portion of the gradient. Co-expression of ORF1p and A3C-WT or C97S/C100S mutant protein shifted ORF1p to fractions 5, 6 and 7 (13–19% sucrose) in which ORF1p and A3C-WT or C97S/C100S mutant containing complexes were detectable. Cell lysates from 2102EP cells expressing endogenous L1 ORF1p were loaded as positive control for ORF1p expression.

To explore the hypothesis that human A3C proteins exhibit their L1-restricting effect after they associate with components of the L1 RNP complex, HeLa cells were co-transfected with L1 reporter plasmid pJM101/L1_RP_ and expression plasmids for A3C-WT or its mutants W74A, R122A or C97S/C100S. Whole-cell lysates of the co-transfected HeLa cells were analyzed by centrifugation in 10–50% continuous sucrose gradients in the presence of Mg^2+^. To identify fractions containing ORF1p and/or HA-tagged A3C-WT or mutant proteins, the 12 fractions of each gradient were analyzed by immunoblotting with anti-L1-ORF1p and anti-HA antibodies (Figure 5). Consistent with a previous report (62), ORF1p was detected throughout the gradient in the absence of ectopically expressed A3C, although the majority of ORF1 proteins formed high-molecular-mass (HMM) complexes, tracked with ribosomal S6 protein (Figure 5). Ectopic co-expression of ORF1p and A3C-WT resulted in a shift of ORF1p to fractions 5–7, which cover the upper portion of the gradient (Figure 5). Co-expression of ORF1p and the CDA double-mutant C97S/C100S, which inhibits L1 transposition to the same degree as A3C-WT, led to the same shift of ORF1p in the gradient. In contrast, in extracts of cells co-transfected with the A3C mutants W74A or R122A, which do not have any L1-restricting effect, the majority of ORF1p signal accumulated in fraction 1 characterized by the highest sucrose concentration of the gradient (Figure 5). Overexpressed A3C-WT or its mutant proteins were detected throughout the gradient, irrespective of whether ectopically expressed ORF1p was present (Figure 5) or not (data not shown). A shift in L1-ORF1p-containing complexes to fractions of lower molecular mass only when A3C-WT or mutant C97S/C100S is present, is consistent with an interaction of A3C-WT or C97S/C100S with either ORF1p alone or the L1 RNP complex and suggests that both proteins are part of complexes that purify mostly in fractions 5–7. The shift could be explained by the interference of A3C proteins with ORF1p multimer-based HMM complexes by an interaction between A3C and ORF1p.

### A3C mutations interfere with the co-localization of ORF1p and A3C-WT in cytoplasmic granules

If co-fractionation of ORF1p and A3C is based on an interaction between both proteins, cellular co-localization of both proteins at some stage during the L1 replication cycle would be expected. To further investigate whether ORF1p and A3C-WT interact with each other, we next tested for co-localization of both proteins in HeLa and 143Btk- cells, which are both known to support retrotransposition of engineered human L1 reporter constructs (75,76). For that purpose, HeLa and 143Btk- cells were co-transfected with the A3C-WT expression plasmid and the reporter construct pDK101, which carries an L1 retrotransposition reporter cassette coding for a T7-tagged L1 ORF1p (62).

**Figure 6.**
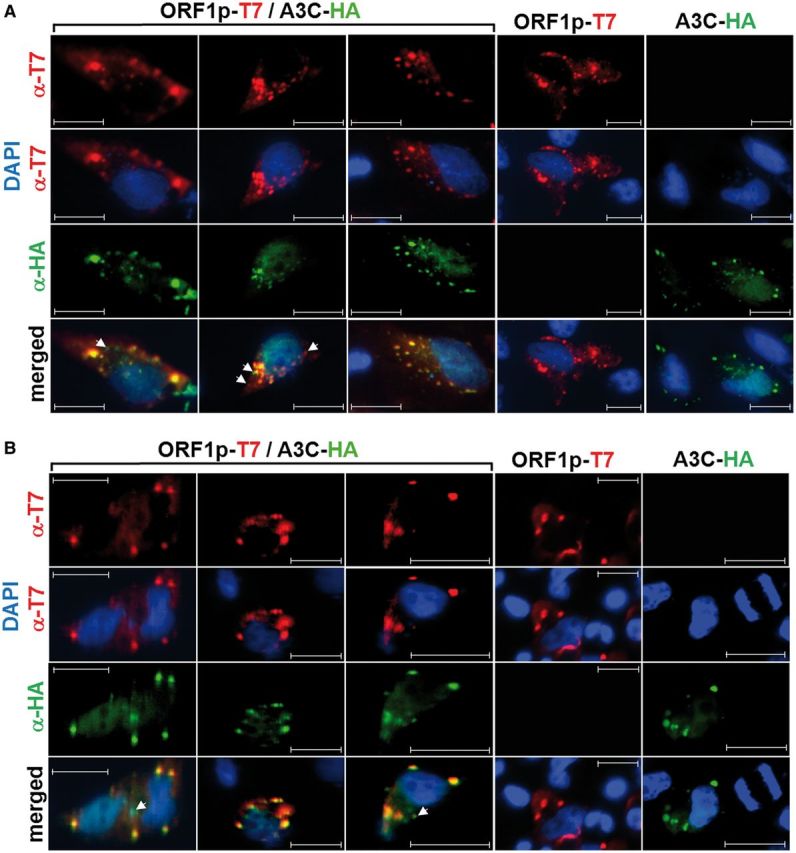

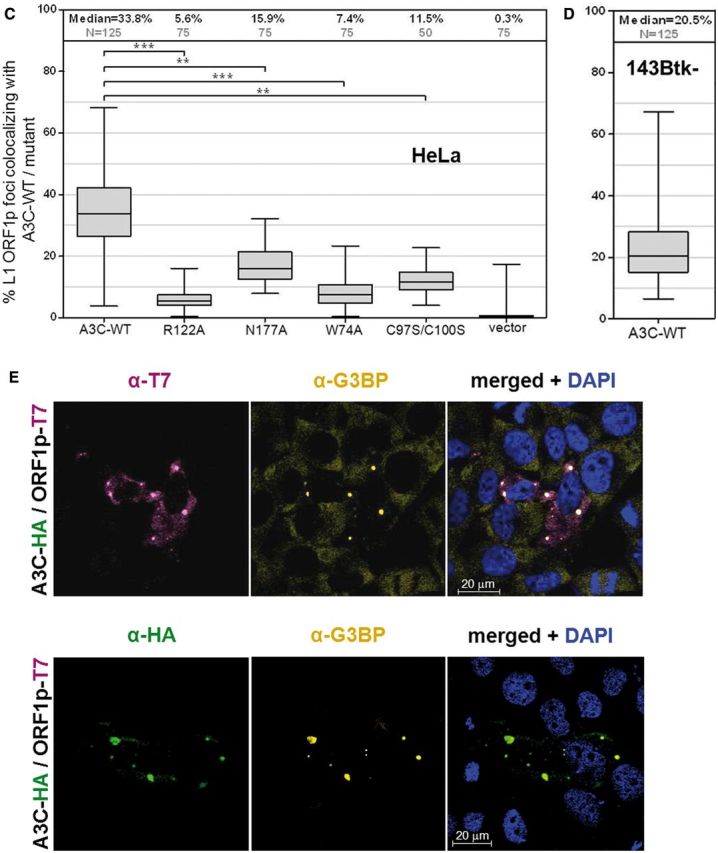
Ectopically co-expressed L1 ORF1p and A3C-WT co-localize in cytoplasmic granules of HeLa and 143Btk- cells. Subcellular localization of T7-tagged L1 ORF1p and HA-tagged A3C-WT proteins in HeLa (**A**) and 143Btk- (**B**) cells was determined by immunofluorescence analysis. ORF1p-T7 and A3C-HA were either co-expressed (ORF1p-T7/A3C-HA) or separately expressed (ORF1p-T7 or A3C-HA). ORF1p-T7 and A3C-HA were stained using α-T7- (red) and α-HA-tag (green) antibodies, respectively. White arrowheads indicate A3C-HA foci or ORF1p granules that do not co-localize with ORF1p granules or A3C-HA foci. Nuclei were visualized with DAPI. Scale bar, 20 µm. (**C**) Mutations in the CDA, dimerization and RNA-binding pocket domains reduce the incidence of co-localization of L1 ORF1p and A3C foci in HeLa cells significantly by variable degrees. Presented are Box-and-Whisker plots of the percentages of ORF1p foci in ORF1p and A3C WT or mutant proteins co-expressing cells that co-localize with HA-tagged WT or mutant A3C foci. Percentages of ORF1p foci co-localizing with mutants of RNA-binding pocket (R122A, N177A), dimerization domain (W74A) and CDA domain (C97S/C100S) are presented. Co-localization of both proteins is most prominent in the case of A3C-WT and absent after transfection with the empty A3C expression vector pcDNA3.1/Zeo(+) (vector). Co-localization of each mutant protein with ORF1p is significantly reduced relative to A3C-WT, but dropped most severely after co-expression with R122A and W74A. N, number of analyzed images, each carrying 10–13 cells, which ectopically co-express ORF1p and A3C-WT or mutant proteins. (**D**) Box-and-Whisker plot of the percentages of ORF1p foci that co-localize with HA-tagged A3C-WT foci in ORF1p and A3C-WT co-expressing 143Btk- cells. (**E**) ORF1p and A3C co-localize with stress granule marker protein G3BP. T7-tagged ORF1p encoded by L1 reporter plasmid pDK101 was co-expressed with HA-tagged A3C in 143Btk- cells (A3C-HA/ORF1p-T7). Co-localization of L1 ORF1p with stress granules was shown by immunofluorescence staining using α-T7 (purple) and α-G3BP (yellow) antibodies, respectively (upper panel). Co-localization of A3C with stress granules was demonstrated separately by confocal microscopy using α-HA (green) and α-G3BP (yellow) antibodies, respectively (lower panel). Nuclei were counterstained using DAPI.

By applying confocal immunofluorescence microscopy, we demonstrate, in accordance with previous reports, that ectopically expressed ORF1p localizes predominantly to cytoplasmic granules in HeLa and 143Btk- cells (Figure 6A and B), which were recently identified as stress granules (77–79). Furthermore, we confirmed nucleocytoplasmic distribution of ectopically expressed A3C-HA proteins in HeLa and 143Btk- cells (37,46) (Figure 6A and B). We found that by far the majority of A3C-HA proteins accumulated in cytoplasmic foci, which we identified in ≥90% of A3C-HA-expressing HeLa cells and is consistent with a previous report (80). Only a much smaller amount was located equally distributed in the nucleus (Figure 6A and B). While it is well established that endogenous as well as ectopically expressed ORF1p nucleates the formation of cytoplasmic granules (78,79,81), it is, to our knowledge, currently unknown whether endogenous A3C accumulates in subcellular compartments. Therefore, we cannot exclude the possibility that the observed formation of cytoplasmic A3C foci is a consequence of A3C overexpression. We found that co-expression of the tagged A3C and ORF1 proteins resulted in a predominant cytoplasmic localization of both proteins in cytoplasmic granules (Figure 6A and B; ORF1p-T7/A3C-HA) in HeLa and 143Btk- cells. Quantitative analyses showed that ∼34% (∼21%) of all cytoplasmic ORF1p granules in those HeLa (143Btk-) cells that co-express ORF1p and A3C-WT ectopically co-localize with A3C-WT foci (Figure 6C and D).

To challenge the hypothesis that an interaction between A3C and ORF1p was responsible for the co-localization of both proteins in the same cytoplasmic granules, we evaluated whether critical mutations in the CDA, dimerization and RNA-binding-pocket domains of A3C affect A3C/ORF1p co-localization. Quantification of the percentage of ORF1p foci co-localizing with A3C mutant proteins (Figure 6C, Supplementary Table S2 and Supplementary Figure S4) showed that the incidence of co-localization drops significantly to ∼5.6–15.9% for the A3C mutant proteins tested. Interestingly, the most prominent drop (by 83 and 78%) was observed for the R122A and W74A mutations, respectively, which were both shown earlier to abolish the L1-restricting effect of A3C entirely (Figures 3 and 4).

Data are consistent with an interaction between A3C-WT and ORF1p, which might direct both proteins to the same cytoplasmic compartment. Results suggest that this interaction may be disturbed by specific mutations introduced into the A3C domains that significantly reduce the incidence of co-localization of ORF1p and A3C foci by variable degrees.

### Both ORF1p and A3C cytoplasmic foci co-localize with stress granule marker G3BP

Since we observed that A3C foci co-localize with ORF1p granules in the cytoplasmic compartment (Figure 6A and B), and endogenous as well as ectopically expressed ORF1p was previously demonstrated to nucleate the formation of stress granules (78,79), we hypothesized that both ORF1p and A3C are directed to the same stress granules. To confirm that the cytoplasmic foci in which ORF1p and A3C co-localize (Figure 6A and B) represent stress granules, we tested whether ORF1p and A3C separately localize to stress granule marker G3BP. To this end, 143Btk- cells were co-transfected with L1 reporter plasmid pDK101 and A3C expression plasmid. ORF1p, A3C and the stress granule–specific marker G3BP were visualized by immunofluorescence microscopy using an α-T7 tag, α-HA tag and an α-G3BP antibody, respectively (Figure 6E). We found that ∼65% of HA-tagged A3C-expressing cells and ≥90% of ORF1p-expressing cells were characterized by detectable cytoplasmic foci. In our double-labeling experiments, 73% of the cytoplasmic A3C foci, and 78% of the ORF1p foci, co-stain with the stress granule marker. Co-localization of cytoplasmic ORF1p foci with G3BP (Figure 6E) confirms the recent finding that ORF1p is found in stress granules (78). The observation that the majority of stress granules in the cytoplasm of co-transfected 143Btk- cells also co-localize with A3C foci (Figure 6E) would be consistent with the sequestration of potential ORF1p/A3C complexes in stress granules. However, not all visible ORF1p foci nor all A3C foci were associated with stress granules (Figure 6E). This could be explained by the dynamic processes involved in building and maintaining RNA-containing compartments like stress granules. Unfortunately, co-staining of the same cell culture sample/foci for all three proteins (ORF1p, A3C and G3BP) was not possible owing to objective technical restrictions. Co-staining with the processing (P) body marker RCK/p54 (82) did not uncover any co-localization of A3C or L1 ORF1p with P-bodies in immunofluorescence experiments (data not shown).

### L1 ORF1p interacts with A3A and A3C

**Figure 7.**
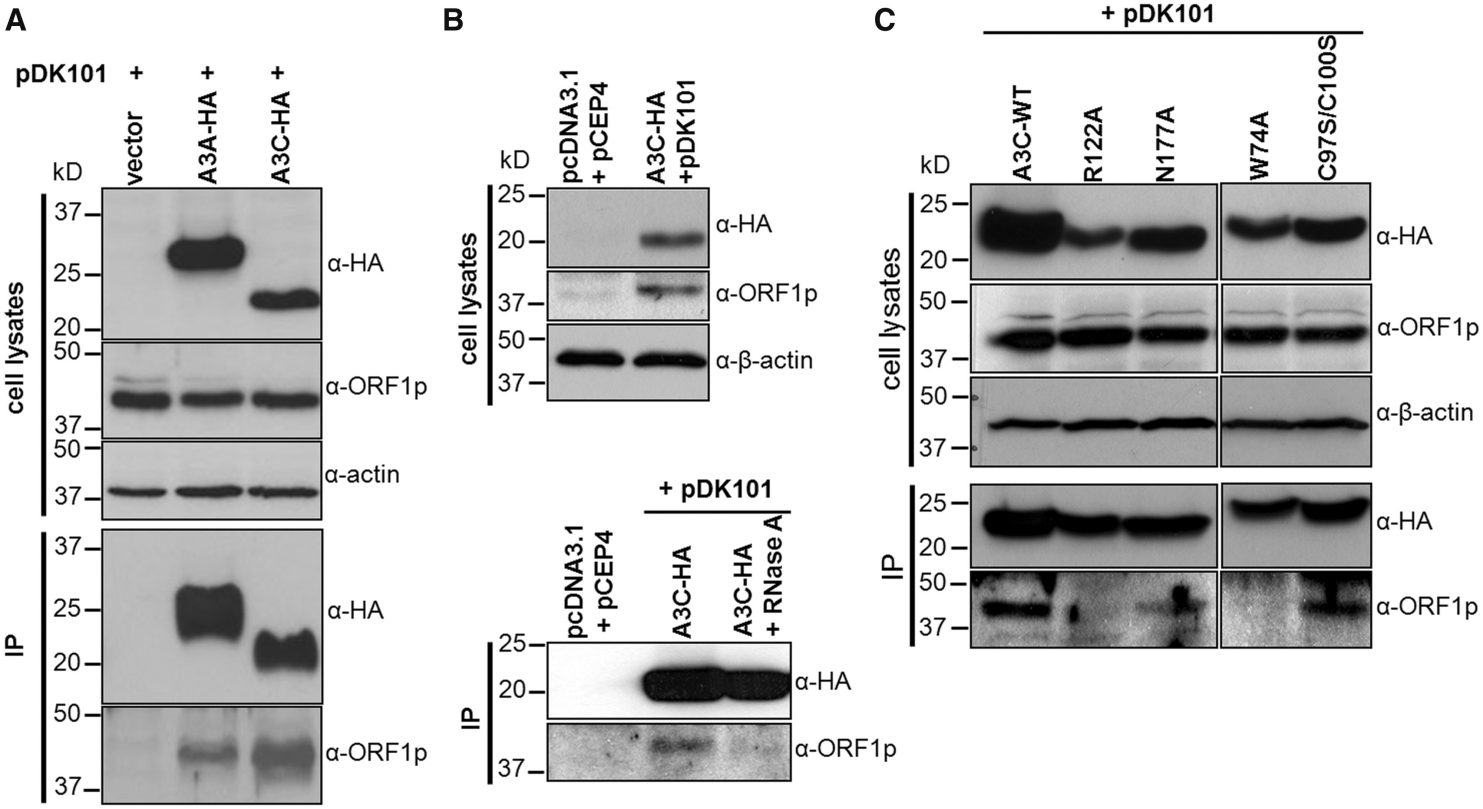
Complex formation between L1 ORF1p and A3C requires bridging RNA molecules. (**A**) ORF1p coprecipitates with ectopically expressed A3A and A3C. Immunoblot analysis of cell lysates isolated from HeLa cells that were consecutively transfected with pDK101 and A3A-HA, A3C-HA or the empty expression vector pcDNA3.1/Zeo(+) (vector), respectively, and their derived immunoprecipitates. The presence of HA-tagged A3A and A3C or L1 ORF1p in cell lysates and immunoprecipitates (IP) was demonstrated using α-HA and α-ORF1p antibodies, respectively. β-actin expression served as loading control. (**B**) RNase A treatment of the cell lysate abolishes the interaction between A3C and L1 ORF1p. Immunoblot analysis of cell lysates isolated from HeLa cells that were co-transfected with pDK101 and A3C-HA or the empty expression vectors (pcDNA3.1 + pCEP4), respectively (upper panel). The cleared cell lysate was split in half and RNase A was added to one half to a final concentration of 50 µg/ml. After incubation of RNase-treated and untreated lysates with α-HA Affinity Matrix Beads, immunoprecipitates were submitted to immunoblot analyses (lower panel). (**C**) RNA-binding pocket mutation R122A abolishes the A3C/L1 ORF1p interaction. Immunoblot analysis of cell lysates isolated from HeLa cells that were co-transfected with pDK101 and expression plasmids encoding A3C-WT, R122A, N177A or C97S/C100S mutant proteins, respectively (upper panel, cell types), and their derived immunoprecipitates (lower panel, IP). In contrast to R122A, RNA-binding pocket mutation N177A does only moderately affect A3C/ORF1p complex formation. While mutating the A3C dimerization site (W74A) abolishes A3C/ORF1p interaction almost entirely, the CDA double-mutant C97S/C100S still exhibits significant interaction with L1 ORF1p. Lysates and immunoprecipitates obtained from W74A- and C97S/C100S-expressing HeLa cells were loaded on a different gel than the remaining lysates and immunoprecipitates.

The shift of ORF1p to sucrose gradient fractions of lower molecular mass if A3C is present (Figure 5), and cytoplasmic co-localization of ORF1p and A3C in human cells (Figure 6), suggests that A3C interacts with ORF1p alone or as part of the L1 RNP complex. To elucidate whether A3C and ORF1p physically interact with each other, HeLa cells were transfected with the L1 reporter plasmid pDK101. After 11 days of hygromycin selection for the presence of the L1 expression plasmid, cells were separately transfected with expression plasmids encoding A3C- and A3A-WT or A3C mutants R122A, N177A, W74A or C97S/C100S. Three days later, cell lysates were isolated and HA-tagged A3 expression as well as L1 ORF1p expression was controlled for by immunoblot analysis (Figure 7A, cell lysates). HA-tagged A3 proteins were immunoprecipitated using anti-HA affinity matrix beads. Efficient immunoprecipitation (IP) was controlled for by immunoblot analysis of the precipitated proteins with the α-HA antibody (Figure 7A, IP). ORF1p was pulled down only if overexpressed A3A or A3C was present, while ORF1p was not precipitated in the absence of APOBEC3 proteins (Figure 7A). To test whether the observed ORF1p-A3C interaction is RNA-bridged, we added RNase A to an aliquot of the cleared cell lysate before IP was performed. RNaseA treatment clearly abolished the interaction between A3C and ORF1p (Figure 7B), suggesting that this interaction is RNA bridged. The finding that the RNA-binding pocket mutation R122A abolishes the A3C-ORF1p interaction (Figure 7C) further supports the existence of an RNA-bridged interaction between A3C-WT and ORF1p. Candidate RNAs that might form an RNA bridge are 5S, 5.8S and/or 7SL RNA, which were demonstrated to interact with A3C-WT, while the R122A mutant was only able to weakly interact with 7SL RNA [Supplementary Figure S5; (63)]. In contrast, the RNA-binding pocket mutation N177A has only a minor effect on A3C/ORF1p interaction. While the CDA domain double-mutation C97S/C100S does not affect the interaction with ORF1p, the W74A mutation in the dimerization domain abolishes the A3C/ORF1p interaction, suggesting that A3C dimerization is required (Figure 7C). Co-immunoprecipitation data are consistent with the percentage of co-localization of WT and mutant A3C proteins with L1 ORF1p (Figure 6).

Taken together, these data suggest an interaction between L1 ORF1p and A3C dimers, which requires RNA binding to A3C. Since both A3C-WT and the CDA domain double-mutant interact with ORF1p and similarly restrict L1 retrotransposition, the interaction with ORF1p seems to be crucial for L1 restriction by A3C. Results also imply that A3A and A3C are incorporated into L1 RNPs via ORF1p interaction.

### The presence of A3C inhibits L1 RT activity

A3G has been reported to be incorporated into budding HIV-1 particles and to inhibit HIV-1 replication by blocking initiation of reverse transcription by the tRNA^Lys3^ primer, physically interacting with the RT, and interacting with the HIV integrase, which was shown to prevent the assembly of a functional pre-integration complex (83–86). Therefore, we next tested the hypothesis that the interaction of A3C with ORF1p and/or L1 RNPs inhibits L1 retrotransposition by interfering with the target-primed reverse transcription of L1 mRNA. For that purpose, we applied the LEAP assay that was established recently to mimic the initial stages of target-primed reverse transcription, where the L1 RT acts to extend a 3′-hydroxyl that has been liberated by the L1 endonuclease (71).

**Figure 8.**
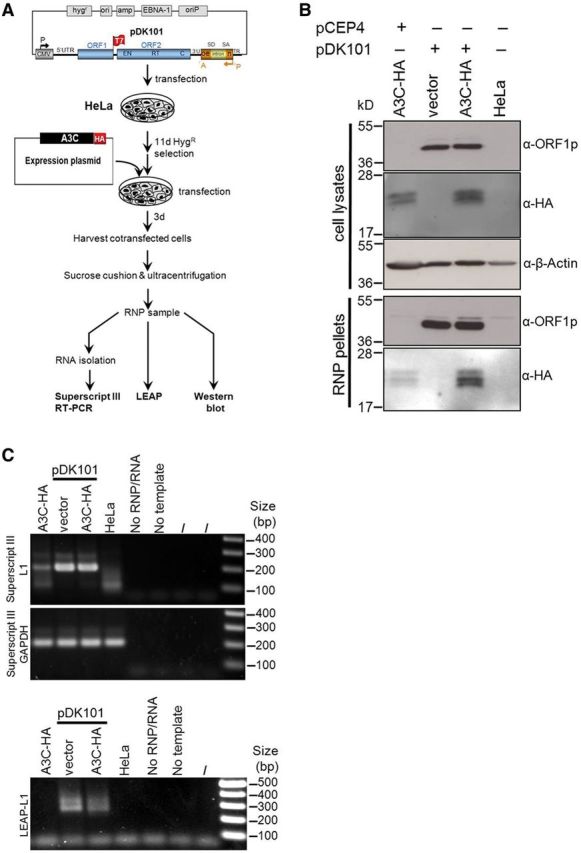
L1 RNPs that assembled in the presence of overexpressed A3C exhibit reduced L1 RT activity. (**A**) Flow chart of the experimental setup. HeLa cells were transfected with the L1 reporter plasmid pDK101 or its empty vector pCEP4, hyg^R^-selected for 11 days and subsequently transfected with the A3C-HA expression plasmid or its empty vector plasmid pcDNA3.1/Zeo(+) (vector). Three days after the last transfection, RNPs were isolated from the lysed cells. Total cell lysates and RNP pellets were obtained from cells consecutively transfected with pCEP4 and the A3C-HA plasmid, pDK101 and A3C-HA or pDK101 and pcDNA3.1/Zeo(+) (vector), and from untransfected cells (HeLa). Subsequently cell lysates and RNP pellets were submitted to immunoblot analysis with α-HA and α-ORF1p antibodies. (**B**) Immunoblot analysis of cell lysates and RNP pellets isolated from HeLa cells overexpressing the L1 protein machinery and/or A3C-HA. β-actin expression served as loading control. Cell lysates and RNP pellets isolated from untransfected HeLa cells and after co-transfection of pDK101 and the empty expression plasmid (vector) served as negative controls. (**C**) LEAP assay to detect L1-specific RT activity. LEAP products are detectable using RNPs from cells, which were co-transfected with pDK101 and A3C-HA or the empty expression vector. Reactions with RNPs from untransfected HeLa cells (HeLa), without template (No template) or RNPs (No RNP/RNA) were used as negative controls. Upper gel: The 207-bp L1-specific SuperScriptIII RT-PCR product is highly abundant in samples from pDK101-transfected cells and absent from HeLa samples. Middle gel: the 198-bp GAPDH SuperScriptIII RT-PCR product is a cellular mRNA marker for all RNP preparations and confirms comparable amounts of RNPs per sample. Lower gel: LEAP products (LEAP-L1).

We set out to compare the L1 RT activity of L1 RNPs that assembled in the presence and absence of overexpressed A3C. For that purpose, HeLa cells were transfected with the L1 reporter plasmid pDK101 or its parental empty vector pCEP4 (Invitrogen), and were then hygromycin selected for the presence of the respective plasmid for 11 days (Figure 8A). Then, hyg^R^-selected cells were transfected with the A3C-expressing plasmid or its empty expression vector pcDNA3.1/Zeo(+). Three days later, the cleared cell lysate was centrifuged through a sucrose cushion and the resultant L1 RNP–containing pellet was resuspended (71). Whole-cell lysates and resuspended L1 RNP–containing pellets were assayed for the presence of ectopically expressed ORF1p and A3C-HA proteins (Figure 8B). Immunoblot analyses showed that similar amounts of ORF1p are both expressed in HeLa cells that were co-transfected with pDK101 (Figure 8B, upper panel) and present in associated RNP pellets (Figure 8B, lower panel). Comparable amounts of A3C-HA were detectable between cell lysates from HeLa cells co-transfected with A3C-HA and pDK101 expression plasmid or with the A3C-HA expression plasmid and the empty pCEP4 expression vector (Figure 8B, upper panel, α-HA). However, only those RNP pellets contain significant amounts of A3C-HA that were formed in the presence of ectopically expressed L1 ORF1p encoded by the transfected pDK101 plasmid (Figure 8B, lower panel, α-HA). In contrast, only negligible amounts of A3C-HA were detectable in RNP pellets from HeLa cells that were not co-transfected with pDK101 and expressed exclusively minor amounts of endogenous L1 ORF1p. The detection of A3C in the RNP pellet is consistent with the incorporation of A3C into L1 RNPs (Figure 8B).

Analysis of RNP pellets by qualitative RT-PCR using SuperScript^TM^ III RT (Figure 8C, upper gel, Superscript III L1) and the same oligonucleotides as primers that were used in the LEAP assay (Figure 8C, lower gel, LEAP-L1), demonstrates the presence of L1 mRNA, which is indicative for the presence of functional L1 RNPs. The RT-PCR experiment indicated that L1 RNA was present at comparable levels in the RNP fraction of HeLa cells that were co-transfected with the empty expression vector and those co-transfected with the A3C-HA expression plasmid. Sequence analysis of the 207-bp SuperScript III RT-PCR product obtained from cells transfected with pDK101 (Figure 8C, upper gel) confirmed the presence of the 3′ end of the pDK101-encoded polyadenylated L1 message. Sequencing of the minor amounts of an unexpectedly observed ∼200-bp RT-PCR product obtained from HeLa cells that were consecutively transfected with the empty expression vector pCEP4 and the A3C-HA expression plasmid (Figure 8C, upper gel, lane A3C-HA) showed that this PCR product was a consequence of unspecific annealing of the PCR primer *L1 3*′ *end* (62) between positions 650 and 680 of the pCEP4 sequence, which is part of the CMV-promoter–controlled transcribed region (data not shown).

Next, we applied the LEAP assay to determine whether those isolated RNPs contained L1-specific RT activity and whether the associated A3C proteins interfere with L1 RT activity. Consistent with previous studies (71,87), a diffuse set of LEAP products that ranged in size from ∼220 to 400 bp was detected in RNPs isolated from HeLa cells that were co-transfected with pDK101 and A3C expression plasmid or with the empty expression vector (Figure 8C, lower gel). Quantification of the LEAP products obtained from the two RNP samples showed that the presence of A3C reduced the amount of L1 cDNA by ∼50% (Figure 8C, lower gel). To evaluate which A3C domains are required for A3C-mediated inhibition of L1 RT, we also performed LEAP assays with RNP fractions carrying the A3C mutant proteins R122A, W74A or C97S/C100S or A3C-WT in parallel. We were able to express reasonable amounts of WT and mutant A3C proteins in HeLa cells, and could demonstrate the presence of A3C-WT in the L1 RNP fraction from HeLa cells co-expressing A3C-WT and pDK101. However, we could not identify any of the mutant A3C proteins R122A, W74A or C97S/C100S in the respective L1 RNP fractions isolated from HeLa cells expressing sufficient amounts of the different A3C mutant proteins (Supplementary Figure S6) and were therefore not able to evaluate any potential effect of the A3C mutants on L1 RNP–associated L1 RT activity. The absence of R122A and W74A from the RNP fractions is consistent with their inability to interact with L1 ORF1p (Figure 7C).

Taken together, our data suggest that A3C-mediated L1 restriction requires an RNA-bridged interaction between A3C dimers and ORF1p of L1 RNP complexes that interfere with the processivity of L1 RT.

## DISCUSSION

Higher eukaryotes have evolved various defense mechanisms to protect their genomes from potential mutagenic effects caused by uncontrolled proliferation of transposable elements. Members of the A3 protein family were reported to inhibit L1 and/or *Alu* retrotransposition by 30–99% [reviewed in (23,74)]. However, no direct evidence for the inhibition of the currently mobilized L1Hs subfamily by cytidine deamination of L1Hs cDNA or any kind of editing of L1 nucleic acid sequences has yet been reported. This strongly suggests that CDA-independent mechanisms are involved in A3-mediated L1 restriction. Since A3C is the most abundantly expressed A3 protein across a wide range of human tissues and cells, we set out to elucidate the A3C-mediated mechanism of L1 restriction.

### A3C-mediated L1 restriction does not require a catalytically functional CDA domain

Using a set of well-characterized A3C CDA mutants, we showed that CDA-activity of A3C does not play any role in A3C-mediated L1 restriction. This is the first example of an A3C-mediated inhibitory effect that is clearly deaminase independent. We also confirmed the previous finding that in contrast corresponding catalytically defective A3A mutants are largely inactive against L1 (37,43). Similar to enzymatically inactive A3C mutants, catalytically inactive mutants of A3B, A3G and AID also maintain L1 inhibition activity (36,42). It was suggested that A3B accomplishes editing-independent inhibition of retroelement mobility by direct interaction with L1 ORF2p through a common domain that interferes with L1 *cis*- and *Alu trans*-mobilization (36). A3C also modestly inhibits *Alu trans*-mobilization with G-to-A mutations being absent from *de novo Alu* insertions that occurred in the presence of overexpressed A3C (39). This observation is consistent with our results indicating that A3C interferes with L1 RT activity. *Alu* retrotransposition can also be inhibited by A3G WT and catalytically inactive mutants, which also suggests a CDA-independent mechanism for A3G action (64,88). It was shown that A3G sequesters *Alu* RNAs in cytoplasmic HMM complexes, particularly Staufen-containing RNA granules, denying these retroelements access to the nuclear L1 protein machinery (88). This inhibitory mechanism differs from the A3A- and A3B-mediated inhibition of *Alu* retrotransposition, where the A3 proteins alter the activity of the L1 machinery in the nucleus (39).

### A3C dimerization and an intact RNA-binding pocket are required for L1 restriction by A3C

To answer the question of whether A3C needs to oligomerize to inhibit L1 mobilization, the effects of the dimerization-deficient A3C mutants F55A and W74A on A3C-mediated L1 inhibition were determined. As observed for SIVΔ*vif* replication (63), we found that A3C-oligomerization is required for the restriction of L1 replication. Similarly, dimerization-deficient A3G deletion mutants cannot restrict HIVΔ*vif* replication (70,89,90). Consistently, structure models of AID, A3C and A3G, as well as the APOBEC2 crystal structure, suggest that APOBEC proteins generally act as dimers or tetramers (63,70,91,92).

While mutating the critical residues K22 or R122 of the putative A3C RNA-binding pocket caused a significant loss of L1 inhibition (p_K22A_ = 0.0015; p_R122A_ = 0.0001) to only ∼29% or even restored and enhanced L1 retrotransposition activity, respectively, the N177A mutation did not affect L1 inhibition by A3C significantly (p_N177A_ = 1). The L1-restricting effect of A3C-WT and its mutant proteins K22A and N177A, as well as the absence of any L1-inhibiting effect of R122A, correlates well with the antiretroviral effects of K22A and N177A and the loss of any inhibitory effect of R122A against SIVΔ*vif* reported previously (63). In contrast to the remaining RNA-binding pocket mutants, R122A abolished the L1-inhibiting effect entirely. It was reported recently that R122A is critically relevant for SIV particle packaging but not for the antiviral activity of A3C, and that the R122A mutation has only a minor inhibiting effect on A3C deaminase activity (63). Furthermore, A3C-WT was shown to interact with 7SL, 5.8S and 5S rRNA, while the R122A mutant maintained only the capability to bind 7SL rRNA [Supplementary Figure S5; (63)]. A strong RNA-bridge candidate is 7SL RNA because it was demonstrated to interact with A3C-WT [Supplementary Figure S5; (63)] and was recently detected in L1 RNP immunoprecipitates (93). Since SRP14, which together with SRP9, forms a complex with 7SL RNA (94), was also reported to be associated with L1 RNPs (93), 7SL RNA could well be an RNA that the ORF1p/A3C interaction depends on.

Our observation that the expression of 0.5–1.5 µg of the mutant R122A expression construct increased the number of L1 retrotransposition events by ∼60–100% can be explained by a potential dominant-negative effect of R122A. Since A3C is endogenously expressed in HeLa cells (37), we hypothesize that R122A mutant proteins oligomerize with intact A3C proteins forming nonfunctional A3C dimers or tetramers, thereby abolishing the L1-inhibiting effect of endogenous A3C-WT. In contrast, expression of the mutant proteins K22A and N177A still restricts L1 activity. We hypothesize that L1 restriction by A3C oligomers requires efficient RNA binding to all involved A3C monomers. This could have the consequence that overexpressed K22A and/or N177A proteins, which were not demonstrated to have any RNA-binding deficiencies, oligomerize with endogenous A3C proteins forming functional RNA-binding oligomers that are still able to restrict L1. This hypothesis is supported by the finding that, in contrast to R122A, the N177A mutant is still able to form complexes with L1 ORF1p (Figure 7C), which requires RNA binding (Figure 7B). Taken together, we conclude that RNA binding to A3C is required for the L1-inhibiting effect of A3C, and that RNA binding to ORF1p is also essential for the observed interaction of A3C with L1 ORF1p. To date, it is unclear which RNA A3C needs to interact with to inhibit L1. Candidate RNAs for A3C interaction are 7SL RNA and 5.8S rRNA, L1 RNA or any host-encoded RNA. Recently it was demonstrated that A3A recognizes and interacts with L1 RNA and is associated with L1 RNA in HMM complexes (46). Interestingly, the ability of A3A to recognize L1 RNA required its catalytic domain. It could well be that the A3C oligomerization that is required for L1 inhibition is RNA dependent. This would be reminiscent of A3G oligomerization that was recently shown to be RNA dependent and required for restriction of HIV-1 (70). It was also reported that, specifically through its RNA-binding properties (17,73,95), A3G sequesters *Alu* and Y RNAs in cytoplasmic HMM complexes away from the nuclear L1 machinery, thereby interdicting the retrotransposition cycle (88).

### A3C interacts with L1 ORF1p and co-localizes with L1 RNPs

Our finding that ORF1p shifts to sucrose gradient fractions of lower molecular mass in the presence of ectopically expressed A3C and the observation that L1 ORF1p and A3C co-localize in HeLa and 143Btk- cells is consistent with an interaction between A3C and ORF1p or L1 RNPs. These data further suggest that A3C and ORF1p are part of HMM complexes (88) and are consistent with the recently reported presence of A3C in HMM complexes of 293T cells (46,96). The physical interaction between ORF1p and A3C or A3A was additionally confirmed by co-immunoprecipitation experiments that strongly imply that both A3 proteins are incorporated into L1 RNPs and exert their inhibiting effects on L1 by interacting with L1 ORF1p or L1 RNPs. Our data strongly suggest that the observed A3C/L1 ORF1p complex results from an RNA-bridged interaction, which means A3C and ORF1p are connected by binding the same RNA molecule (e.g. 7SL RNA). This is further supported by the following facts: (i) both A3C and ORF1p are RNA-binding proteins (63,97,98), and (ii) an intact RNA-binding pocket is required for A3C-mediated restriction of both SIVΔ*vif* and L1 replication. Furthermore, RNA binding was shown to be crucial for the interaction between A3C and SIV-NC (63), and A3B and mA3 were reported to bind to L1 ORF1p via an RNA bridge (99). It is conceivable that A3C binds L1 RNA that is interacting with ORF1p, thereby preventing the nuclear import of L1 RNPs. Taken together, we provide direct evidence for an RNA-bridged association of A3C proteins with L1 ORF1p or L1 RNPs.

### Association of A3C with stress granules

In accordance with previous reports, we demonstrate that overexpressed ORF1p localizes predominantly to cytoplasmic granules in HeLa and 143Btk- cells (Figure 6A and B), which were recently identified as stress granules (77–79). Ectopic co-expression of A3C-HA led to nucleocytoplasmic distribution of A3C-HA proteins and their accumulation in cytoplasmic foci, which is consistent with the A3C distribution observed when A3C is expressed alone (37,46,80). We found that in HeLa cells co-expressing both proteins, 34% of all cytoplasmic ORF1p granules co-localize with A3C-HA foci, suggesting that A3C may block the entry of a subset of L1 RNPs into the nucleus by A3C-mediated sequestration of L1 RNPs. Since our data show that both A3C and L1 ORF1p localize with stress granules, it is most likely that A3C and L1 ORF1p also co-localize at least in a subset of stress granules. Our findings are consistent with the recent identification of L1 ORF1p together with polyadenylated RNA in stress granules (78,87). Co-localization of A3C and L1 ORF1p in stress granules would support the hypothesis that A3C mediates the transport of functional L1 RNPs into stress granules. It is also consistent with a recently proposed mechanism whereby the cell may mitigate the potential mutagenic effects of retrotransposition by sequestering L1 RNPs and possibly targeting them for degradation (78). While we could not find any evidence for A3C localization to P-bodies, A3G, A3B and A3F were reported to be located to P-bodies (88,100–102).

### A3C interferes with L1 RT activity

Since our data suggest the incorporation of A3C into L1 RNPs, we tested whether the L1 RT activity of L1 RNPs that are associated with A3C is impaired. We found that the presence of A3C led to a decrease of L1 cDNAs by ∼50%, which roughly corresponds to the reduction of the number of detectable L1 retrotransposition events in HeLa cells by ∼50–75% when overexpressed A3C is present. This is reminiscent of the nonenzymatic form of inhibition of HIV-1Δ*vif* infectivity by A3G, which likely involves physical impairment of RT activity (73,86,103). Taken together, we uncovered a novel deaminase-independent mechanism of L1 inhibition by A3C that is mediated by the interaction of A3C with L1 ORF1p, requires the binding of RNA, and interferes with L1 RT activity. Impairment of L1 RT activity not only inhibits *cis*-mobilization of L1 elements but also *trans*-mobilization of *Alu* and SVA elements as well as processed pseudogene formation. Our data also provide some evidence for an additional mechanism of L1 restriction based on A3C-mediated transport of functional L1 RNPs into stress granules where they might be targeted for degradation. The near-ubiquitous expression of A3C may underline its significant biological role in the repression of potentially deleterious L1-mediated retrotransposition events.

While there is no evidence for A3-mediated editing of members of the currently mobilized L1Hs subfamily, ∼1300 edited members of the L1 subfamilies L1PA2, L1PA3, L1PA5 and L1PA6 were recently identified, covering only ∼0.1% of L1 elements (104). This suggests that A3-mediated editing of L1 elements occurred between 5.6 and 34 Myrs ago (105). Surprisingly, it was also reported that 20.1% of all SVA elements including 238 human-specific elements and 16 polymorphic elements carry G-to-A hypermutations, indicating that, in spite of everything, A3-mediated DNA editing of non-LTR retrotransposons is a still ongoing process in humans (104).

## SUPPLEMENTARY DATA

Supplementary Data are available at NAR Online.

## FUNDING

Deutsche Forschungsgemeinschaft [DA 545/2-1 to G.G.S]; Heinz Ansmann Foundation for AIDS Research (to C.M.); Stiftung zur Erforschung infektiös-immunologischer Erkrankungen‘ (to H.H.). Funding for open access charge: Paul-Ehrlich-Institut.

*Conflict of interest statement*. None declared.

## Supplementary Material

Supplementary Data
