# Supplementary material for: Human LINE-1 restriction by APOBEC3C is deaminase independent and mediated by an ORF1p interaction that affects LINE reverse transcriptase activity
Source: Nucleic Acids Res. 2013 Oct 5;42(1):396–416. doi: 10.1093/nar/gkt898 (PMC3874205; doi:10.1093/nar/gkt898)
Supplement: Supplementary Data [file supp_42_1_396__index.html]

Human LINE-1 restriction by APOBEC3C is deaminase independent and mediated by an ORF1p interaction that affects LINE reverse transcriptase activity — Human LINE-1 restriction by APOBEC3C is deaminase independent and mediated by an ORF1p interaction that affects LINE reverse transcriptase activity — Supplementary Data 

# Human LINE-1 restriction by APOBEC3C is deaminase independent and mediated by an ORF1p interaction that affects LINE reverse transcriptase activity

## Supplementary Data

files

**Files in this Data Supplement:**

- Supplementary Data - pdf file
