# Supplementary material for: Human LINE-1 restriction by APOBEC3C is deaminase independent and mediated by an ORF1p interaction that affects LINE reverse transcriptase activity
Source: Nucleic Acids Res. 2013 Oct 5;42(1):396–416. doi: 10.1093/nar/gkt898 (PMC3874205; doi:10.1093/nar/gkt898)
Supplement: Supplementary Data [file supp_gkt898_nar-02013-h-2013-File011.pdf]

Supplementary Data

Human LINE-1 restriction by APOBEC3C is deaminase independent and mediated by an ORF1p interaction that affects LINE reverse transcriptase activity

Axel V. Horn, Sabine Klawitter, Ulrike Held, André Berger, Ananda Ayyapan Jaguva Vasudevan, Anja Bock, Henning Hofmann, Kay-Martin O. Hanschmann, Jan-Hendrik Trösemeier, Egbert Flory, Robert A. Jabulowsky, Jeffrey S. Han, Johannes Löwer, Roswitha Löwer, Carsten Münk and Gerald G. Schumann

Supplementary Methods

Coimmunoprecipitation and Immunoblot analysis to characterize A3C dimerization

$2x10^5$ 293T cells were cotransfected with 1μg of a plasmid expressing V5-tagged A3C-WT and 1μg expression construct encoding HA-tagged A3C-WT, F55A or W74A, respectively, using Lipofectamine LTX (Invitrogen). Two days after cotransfection, cells were harvested and lysed in ice-cold lysis buffer [25mM Tris (pH 8.0), 137 mM NaCl, 1 % glycerol, 0.1 % SDS, 0.5 % Na-deoxycholate, 1 % Nonidet P-40, 2 mM EDTA, and complete protease inhibitor mixture (Roche)]. Small aliquots of the cleared lysates were subjected to SDS-PAGE followed by transfer to a PVDF membrane. The remaining cleared lysates were incubated with 30 μl α-HA Affinity Matrix Beads (Roche) for 60 min at 4°C. The samples were washed 5 times with ice-cold lysis buffer. Bound proteins were eluted by boiling the beads for 5 min at 95°C in SDS loading buffer.

A3C-HA proteins were detected using an α-HA antibody ($1{:}10^4$ dilution; Covance) and α-mouse horseradish peroxidase (1:7500 dilution; Amersham Biosciences). For detection of A3C-V5, an α-V5 antibody (1: 4000 dilution; Serotec) was applied. Alpha-tubulin was detected using an α-tubulin antibody ($1{:}10^4$ dilution; Sigma). Signals were visualized by ECL plus (Amersham Biosciences).

Analysis of A3C–RNA interaction

To demonstrate protein–RNA interaction, plasmids expressing A3C-WT or R122A were transfected to 293T cells as described above. Cells were lysed in ice-cold lysis buffer

[PBS with 1% Triton X-100, 16 U/mL RiboLock RNase Inhibitor (Fermentas), and complete protease inhibitor mixture (Roche)]. The cleared lysates were incubated with 50µl-HA Affinity Matrix Beads (Roche) for 60 min at 4°C. The samples were washed 5 times with ice-cold lysis buffer. RNA bound to immobilized proteins was extracted from HA beads using TRIzol reagent (Invitrogen), according to the manufacturer's instructions. RNAs coprecipitated with A3C proteins were dissolved in diethyl pyrocarbonate–treated H_2O. Specific RT-PCR on A3C-bound RNAs was performed using Revert Aid First Strand cDNA synthesis kit (Fermentas) and random hexamer primers.

Supplementary Tables

Supplementary Table S1: **L1 retrotransposition rates in the presence of overexpressed WT and mutant A3A and A3C proteins.** WT, pJM101/L1$_{RP}$; RT-, negative control construct pJM105/L1$_{RP}$; N, number of individual cotransfection experiments;

	APOBEC Construct	LINE-1 Status	N	Retrotransposition frequency ($2x10^5$ transfected)	Experimental range ($2x10^5$ transfected)	Wild-type activity (%) ± s.e.m.	p-value (1) (relative to mock)		p-value (2) (relative to A3C-WT)	
Comparison of A3A/A3C CDA Mutants	mock	WT	6	136.8	121-158	100.0 ± 7.7	/	/	/	/
	A3A-WT	WT	6	8.7	6-14	6.3 ± 1.6	<0.0001	***	/	/
	A3A-E72A	WT	6	136.5	116-156	99.8 ± 8.4	1.0000		<0.0001	***
	A3A-C101A/C106A	WT	6	217.7	161-245	159.1 ± 15.4	0.0014	**	<0.0001	***
	A3C-WT	WT	6	40.2	32-44	29.4 ± 3.3	<0.0001	***		
	A3C-E68Q	WT	6	85.8	54-119	62.7 ± 11.1	0.0063	**	0.0054	**
	A3C-C97S/C100S	WT	6	46.8	40-54	34.2 ± 2.6	<0.0001	***	0.3282	
	A3C-WT	RT-	6	1.2	0-4	0.9 ± 0.9	/	/	/	/
A3C CDA Mutants	mock	WT	3	202	177-218	100.0 ± 8.3	/	/	/	/
	A3C-WT	WT	3	91.7	87-100	45.4 ± 2.8	0.0093	**	/	/
	A3C-C97S	WT	3	25.3	18-30	12.5 ± 2.4	0.0014	**	0.0023	**
	A3C-C100S	WT	3	58.7	51-63	29.0 ± 2.5	0.0033	**	0.0349	*
	A3C-H66R	WT	3	102	93-114	50.5 ± 4.0	0.0168	*	1.0	
	A3C-WT	RT-	3	0	0-0	0 ± 0				

A3C Dimerization Mutants	mock	WT	6	136.8	121-158	100.0 ± 7.7	/	/	/	/
	A3C-WT	WT	6	40.2	32-44	29.4 ± 3.3	<0.0001	***	/	/
	A3C-F55A	WT	6	144.2	115-161	105.4 ± 11.3	1.0000		<0.0001	***
	A3C-W74A	WT	6	135.5	112-148	99.0 ± 6.8	1.0000		<0.0001	***
	A3C-WT	RT-	6	1.2	0-4	0.9 ± 0.9				
A3C RNA Binding Pocket Mutants	mock	WT	6	222.3	179-269	100.0 ± 9.6	/	/	/	/
	A3C-WT	WT	6	83.7	60-103	37.6 ± 5.4	<0.0001	***	/	/
	A3C-K22A	WT	6	158	115-196	71.1 ± 9.2	0.0236	*	0.0015	**
	A3C-N177A	WT	6	103.8	38-121	46.7 ± 6.2	0.0002	***	1.0	
	A3C-R122A	WT	6	361.4	231-440	162.6 ± 21.6	0.0328	*	0.0001	***
	A3C-WT	RT-	6	0	0-0	0 ± 0				
Titration of A3C-WT and A3C RNA Binding Pocket Mutant R122A	mock	WT	3	147.3	138-157	100.0 ± 6.5	/	/	/	/
	0.25µg A3C-R122A	WT	3	137	120-153	93.0 ± 11.2	1.0000		/	/
	0.5µg A3C-R122A	WT	3	289.7	241-327	196.6 ± 29.9	0.0546		/	/
	0.75µg A3C-R122A	WT	3	270.3	241-290	183.5 ± 17.6	0.0151	*	0.0008 (3)	***
	1.5µg A3C-R122A	WT	3	256.7	230-303	174.2 ± 27.3	0.1022		0.0086 (4)	**
	0.25µg A3C-WT	WT	3	22.3	18-25	15.2 ± 2.6	0.0003	***	/	/
	0.5µg A3C-WT	WT	3	35.7	20-48	24.2 ± 9.7	0.0035	**	/	/
	1.0µg A3C-WT	WT	3	34.3	22-44	23.3 ± 7.6	0.0018	**	/	/
	1.5µg A3C-WT	WT	3	32	25-41	21.7 ± 5.6	0.0009	***	/	/
	0.5µg A3C-WT	RT-	3	0	0-0	0 ± 0	/	/	/	/

p-values adjusted from t-test according to Bonferroni multiple comparisons

(1) p-values resulting from comparison with retrotransposition rates in mock-transfected cells
(2) p-values resulting from comparison with retrotransposition rates in A3C-WT-transfected cells
(3) p-value resulting from comparison of retrotransposition rates: 0.75 µg A3C-R122A vs. 0.25 µg A3C-WT
(4) p-value resulting from comparison of retrotransposition rates: 1.5 µg A3C-R122A vs. 0.5 µg A3C-WT

Supplementary Table S2: Percentage of L1 ORF1p foci in HA-HeLa and 143 BTK- cells colocalizing with A3C-WT or mutant protein.

Cell type	Mutan	N[1]	Median [%]	Min [%]	LQ [%][2]	UQ [%][2]	Max [%]
HA HeLa	A3C wildtype	125	33.8	3.8	26.8	42.1	68.2
	R122A	75	5.6	0.4	4.0	7.5	16.1
	N177A	75	15.9	7.9	12.5	21.4	32.2
	W74A	75	7.4	0.4	4.7	10.8	23.3
	C975/C100S	50	11.5	4.1	9.3	14.6	22.8
	pcDNA	75	0.3	0.0	0.0	0.8	17.4
143 BTK-	A3C wildtype	125	20.5	6.5	15.2	28.1	67.3

1 – Number of percentages (25 images per plate evaluated); 2 – Lower and Upper Quartile

Supplementary Figures

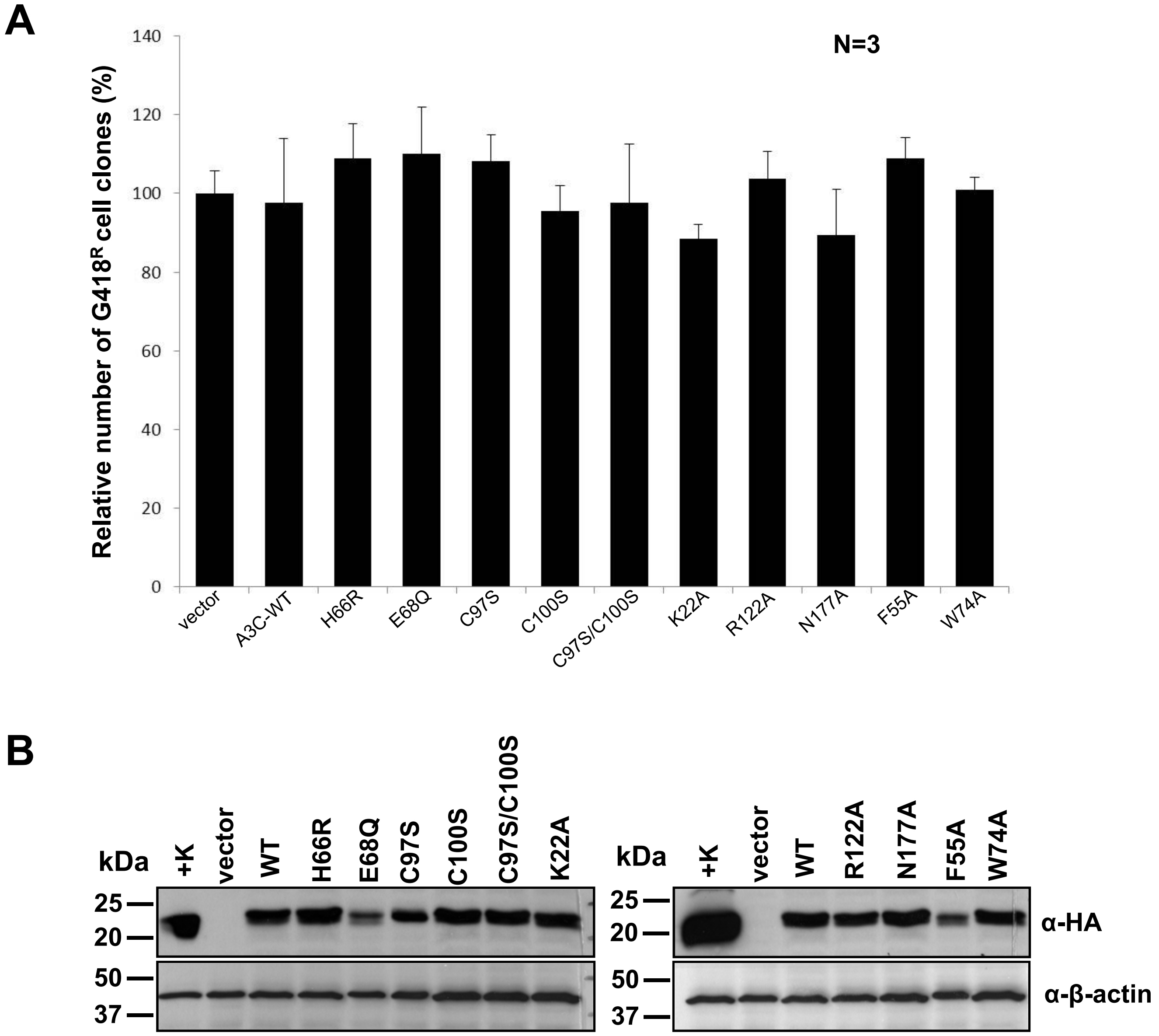

Supplementary Figure S1: Evaluation of potential cytotoxic effects of APOBEC3C and its mutant proteins.

A) Overexpression of WT or mutant A3C proteins has no considerable effect on cell viability. HeLa cells were cotransfected with 0.5 μg of pcDNA3.1(+) and 0.5 μg of each A3C-WT or mutant expression plasmid or parental plasmid pcDNA3.1/Zeo(+) (vector). In the case of the A3C mutant R122A, it was necessary to cotransfect 1.5 μg expression plasmid with 0.5 μg of pcDNA3.1(+) to ensure that the cellular R122A expression level was comparable to the remaining A3C mutant and WT proteins (see B). The number of G418^{R} cell colonies in the absence of any overexpressed A3C-WT or mutant protein was set as 100% (vector). Bars represent arithmetic means SD of three independent cotransfection experiments.

B) Immunoblot analysis of the expression levels of HA-tagged WT and mutant A3C proteins that were existent during the toxicity assay using an anti-HA antibody. β-actin expression served as loading control.

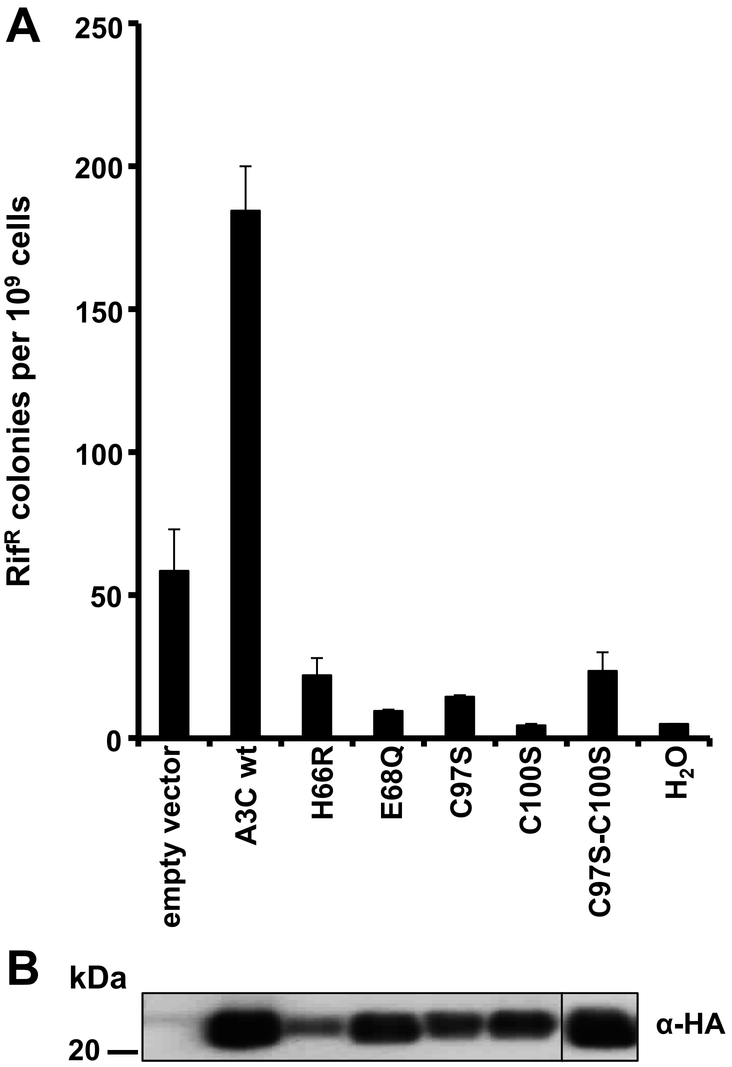

Supplementary Figure S2:
A) Editing activity of A3C-WT and A3C CDA mutant proteins measured as Rif-resistant (Rif^R) colonies. B) Immunoblot analysis to demonstrate expression levels of A3C-WT and mutant proteins in *E. coli*. Cell extract of C97S/C100S double mutant expressing cells was blotted on the same membrane as the remaining samples, but was originally separated from the samples by several lanes.

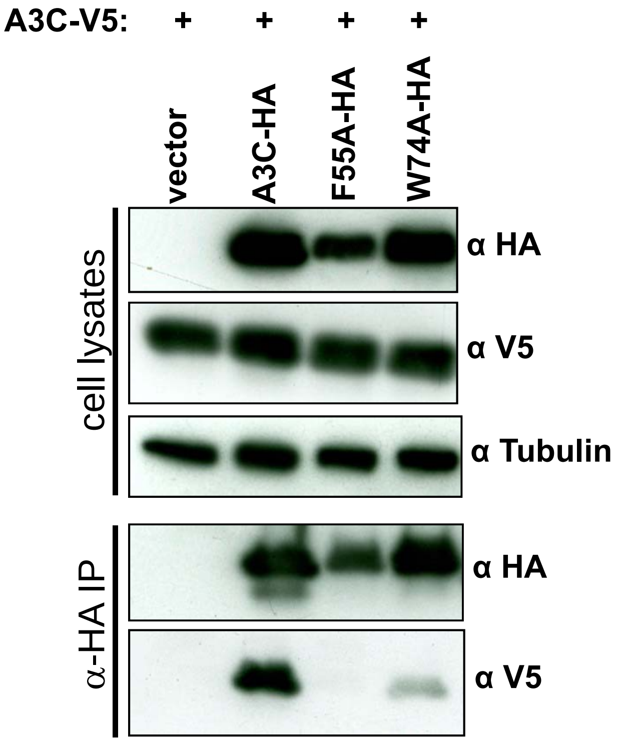

Supplementary Figure S3: Immunoblot analysis of A3C dimerization: F55 and W74 residues participate in the dimerization of A3C. 293T cells were cotransfected with the respective HA-tagged A3C-WT or mutant (F55A and W74A) expression construct and the V5-tagged A3C-WT expression plasmid. Cell lysates and immune precipitates (IP) were subjected to immunoblot analysis using anti-HA- and anti-V5-antibodies, respectively. W74A-HA coprecipitated only negligible amounts of V5-tagged A3C-WT, while F55A-HA was not able to bind any detectable V5-tagged A3C-WT. Tubulin expression served as loading control.

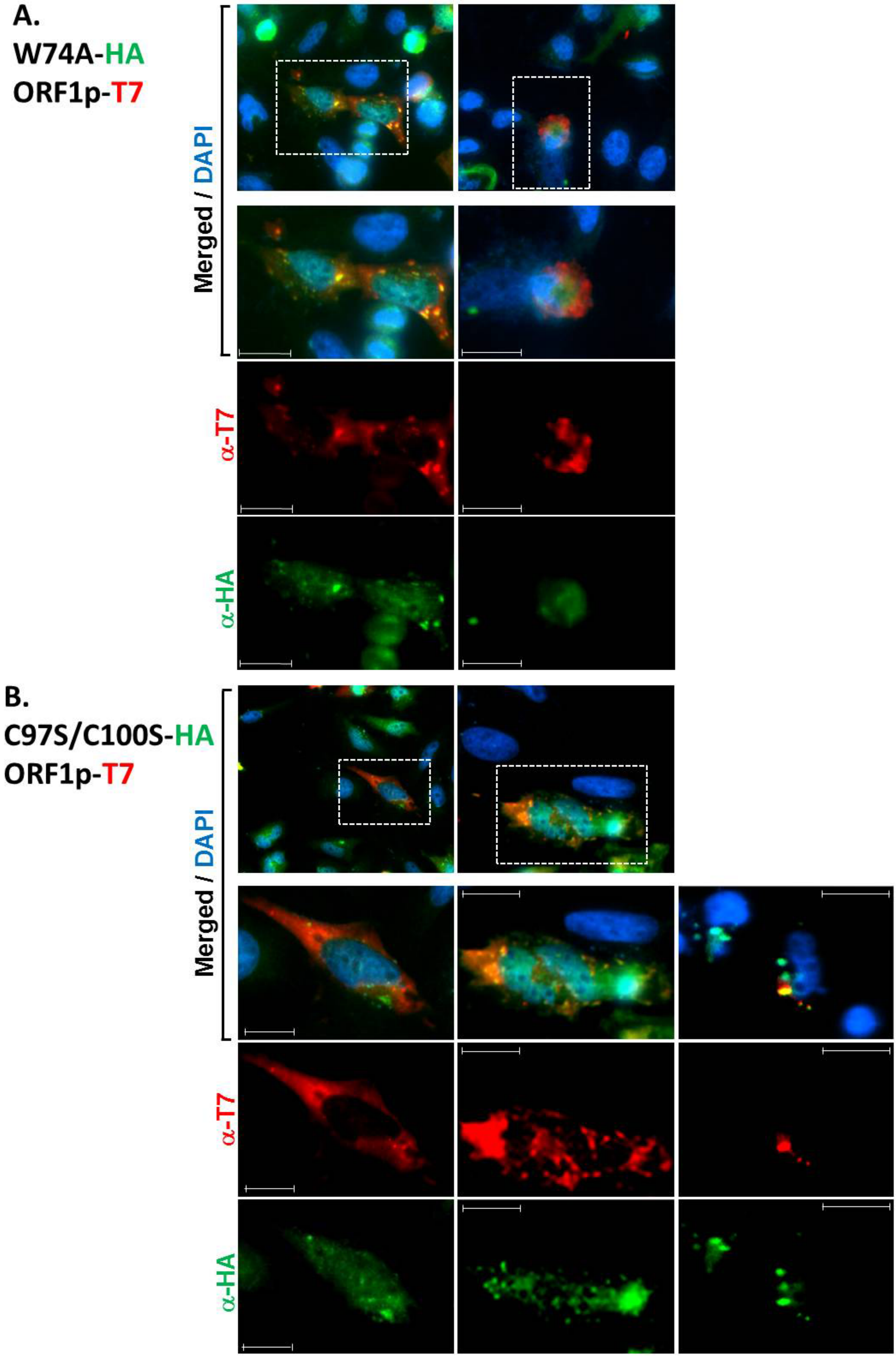

Supplementary Figure S4

C.
R122A-HA
ORF1p-T7

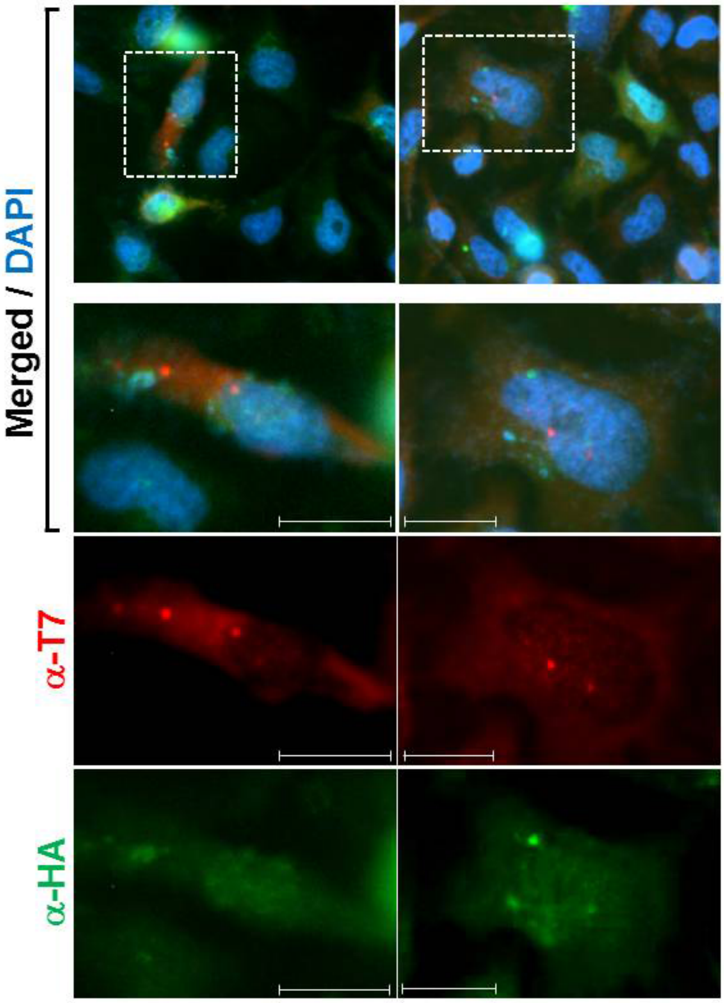

D.
N177A-HA
ORF1p-T7

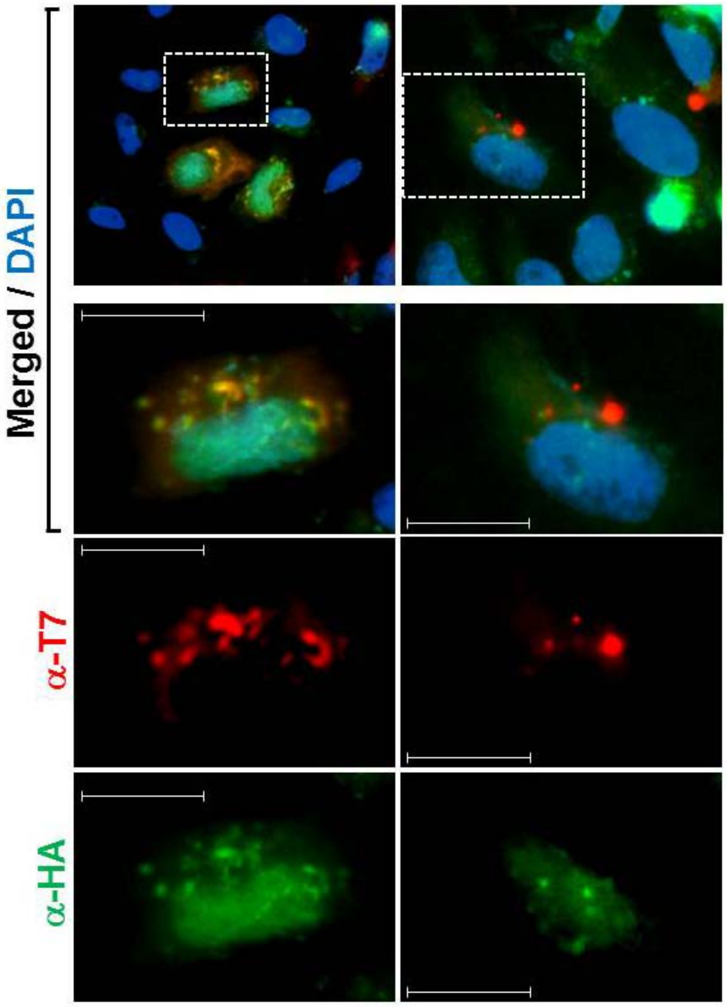

Supplementary Figure S4

Supplementary Figure S4: Subcellular localization of coexpressed T7-tagged L1 ORF1p and HA-tagged A3C mutant proteins W74A (A), C97S/C100S (B), R122A (C), and N177A (D) in HeLa cells. Immunofluorescence staining was performed using α-T7 (red) and α-HA-tag (green) antibodies. Two (W47A, R122A, N177A) or three (C97S/C100S) representative images of stained, cotransfected cells are presented. A3C mutations negatively affect the formation of A3C foci and their frequency of colocalization with ORF1p granules by varying degrees. The quantification of colocalization frequencies is illustrated in Figure 6C.

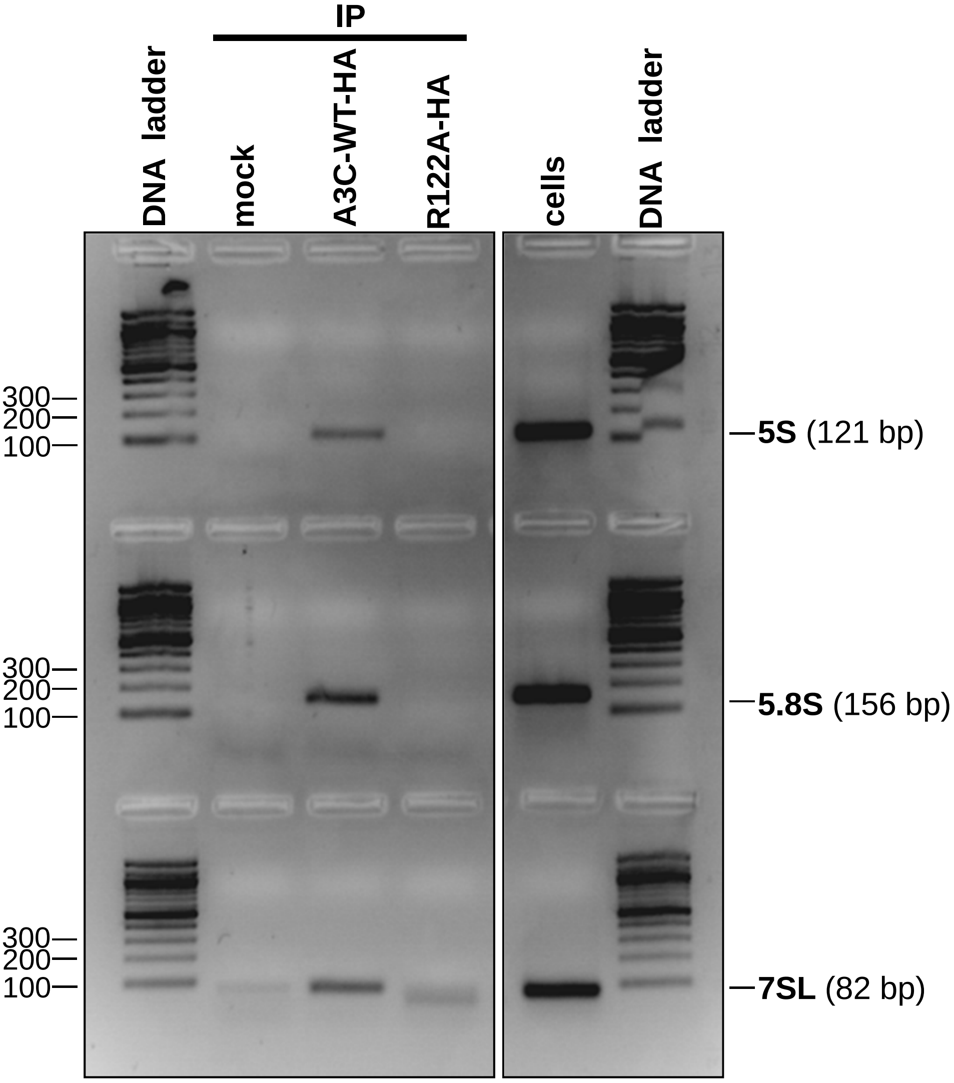

Supplementary Figure S5: RT-PCR on RNA interacting with A3C proteins. Isolated and A3C-bound RNA (IP) was reverse-transcribed and amplified using specific primers for 5S, 5.8S and 7 SL RNA. Background signal was determined with RNA from untransfected cells (mock). Availability of the tested RNAs in the cells was confirmed for each sample through RT-PCR on RNA isolated from cells before immunoprecipitation (IP) was performed (cells). DNA size standard, GeneRuler™ 100-bp Plus DNA Ladder (Fermentas).

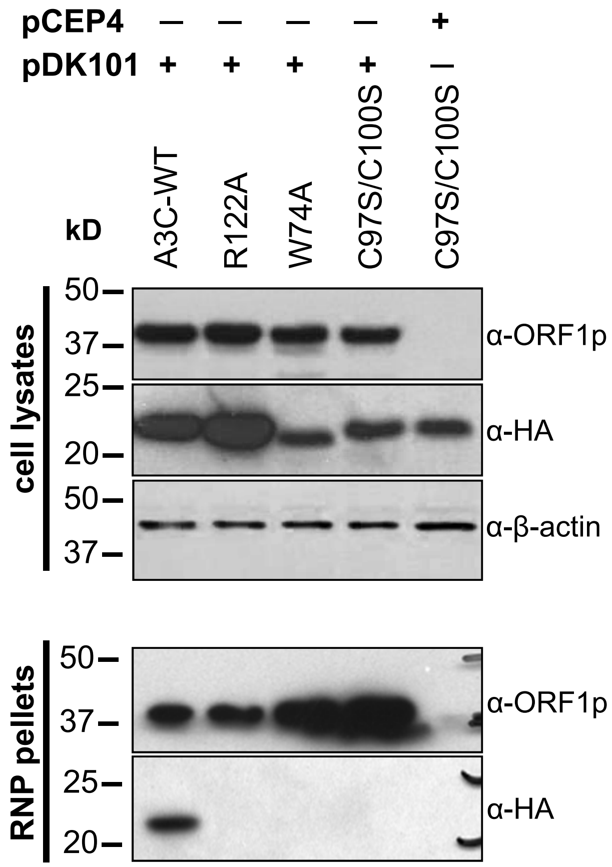

Supplementary Figure S6: Mutant A3C proteins are not associated with L1 RNP fractions. Immunoblot analysis of cell lysates and RNP pellets isolated from HeLa cells overexpressing the L1 protein machinery and HA-tagged A3C-mutants R122A, W74A or C97S/C100S or A3C-WT. β-actin expression served as loading control. Cell lysates and RNP pellets isolated from HeLa cells that were cotransfected with pCEP4 and the C97S/ C100S expression construct, served as negative control for RNP formation. α-HA antibody was used to detect expression of HA-tagged wildtype and mutant A3C proteins.

Data show the expression of large amounts of the L1 protein machinery and A3C-WT and mutant A3C proteins in cotransfected HeLa cells. RNPs isolated from these cells did not include any A3C mutant proteins, although A3C-WT was efficiently incorporated in the RNP fraction (RNP pellets).

While the absence of mutants R122A and W74A from the RNPs is consistent with their inability to interact with L1 ORF1p (Figure 7C), the C97S/C100S double mutant was expected to be part of the RNPs because it was shown to interact with ORF1p (Figure 7C). However, the absence of the C97S/C100S mutant protein from L1 RNPs could be explained by the fact that salt/buffer conditions L1 RNPs are exposed to during the RNP isolation procedure, are not concordant with conditions used for immunoprecipitation experiments presented in Figure 7.
